# Wayfinding with Impaired Vision: Preferences for Cues, Strategies, and Aids (Part I—Perspectives from Visually Impaired Individuals)

**DOI:** 10.3390/brainsci16010013

**Published:** 2025-12-22

**Authors:** Dominique P. H. Blokland, Maartje J. E. van Loef, Nathan van der Stoep, Albert Postma, Krista E. Overvliet

**Affiliations:** 1 Experimental Psychology, Helmholtz Institute, Utrecht University, 3584 CS Utrecht, The Netherlandsa.postma@uu.nl (A.P.); k.e.overvliet@uu.nl (K.E.O.); 2 Human Machine Teaming, Defense, Safety, and Security, Nederlandse Organisatie voor Toegepast-Natuurwetenschappelijk Onderzoek (TNO), 3769 DE Soesterberg, The Netherlands; nathan.vanderstoep@tno.nl

**Keywords:** orientation, mobility, navigation, low vision, visual impairment, blindness, thematic analysis, sensory preferences, wayfinding strategies

## Abstract

People with visual impairments (VIPs) can participate in orientation and mobility (O&M) training to learn how to navigate to their desired goal locations. During O&M training, personal wayfinding preferences with regard to cue use and wayfinding strategy choice are taken into account. However, there is still a lack of clarity about which factors shape VIPs’ wayfinding experiences and how. Background/Objectives: In this study, we mapped individual differences in preferred sensory modality (both orientation- and mobility-related), and classified which personal and environmental factors are relevant for these preferences. Methods: To this end, interviews were conducted with eleven Dutch VIPs whose impairment varied in onset, ontology, and severity. Results: We concluded from our thematic analysis that hearing is the most important sensory modality to VIPs for orientation purposes, although it varies per person how and how often other resources are relied upon (i.e., other sensory modalities, existing knowledge of an environment, help from others, or navigational aids). Additionally, environmental factors such as weather conditions, crowdedness, and familiarity of the environment influence if, how, and which sensory modalities are employed. These preferences and strategies might be mediated by individual differences in priorities and needs pertaining to energy management. Conclusions: We discuss how the current findings could be of interest to orientation and mobility instructors when choosing a training strategy for individual clients.

## 1. Introduction

Navigation is the purposeful act of traveling to and from important destinations within the world, like a workplace or a friend’s home. As such, navigation is an essential part of our everyday lives and critical to independent functioning. Navigation is understood as a combination of two components, orientation and mobility [1,2]. Orientation, or the wayfinding aspect of navigation, involves all the cognitive processes necessary for making the journey. These processes include, but are not limited to, the memorization of routes, spatial decision-making during route-following (e.g., knowing when to take a left or right turn), localization (i.e., determining where one is within a given environment), and landmark recognition [3,4,5,6,7,8]. Mobility, or the locomotion aspect of navigation, on the other hand, involves the immediate interaction of the embodied navigator and the environment [7,9]. Examples of mobility-related skills include avoiding obstacles and managing tripping hazards. For effective orientation and mobility, people rely on various types of self-motion cues and sensory cues from their external environment [10], with the visual modality often considered as an essential source of spatial information [11,12,13,14,15,16]. However, for people with visual impairments (VIPs), visual information is not (fully) accessible, which can make it challenging for them, compared to sighted individuals, to reach their desired destinations safely, independently, and efficiently.

Challenges that VIPs face when navigating public spaces include risk of falling, identifying the correct trains, entrances, or restrooms, taking detours when encountering unexpected constructions, avoiding obstacles like cars parked on sidewalks, traversing Shared Spaces, encountering social stigma or unsafe situations, and safely crossing a street [17,18,19,20,21,22,23,24]. These examples represent only a subset of the many challenges VIPs encounter in their daily navigation, and since the VIP population is quite heterogeneous, it differs per person which of these challenges are more or less present. Understanding the nuances, interplay, and diversity in wayfinding experiences within this population can contribute to further increasing VIPs’ experiences of independence, safety, self-confidence, freedom of movement, and social inclusion [25,26,27,28,29,30]. These experiences are all important for the basic human dignity, quality of life, and mental health that people with disabilities have an inherent right to [31,32], for example, by having a choice regarding the mode of transport or by choosing to leave the house in the first place [18,21].

Because of the challenges associated with vision loss, visually impaired individuals rely on multiple sensory modalities for orientation, often achieving spatial task performance similar to that of sighted individuals (for an overview, see, e.g., [12] or [33]). Of course, these findings should be interpreted with the understanding that the VIP population is not homogenous, and is influenced by factors such as age or the severity and onset of vision loss (see, e.g., [34,35,36,37]). Nevertheless, how VIPs navigate might not only depend on which cues are or are not available to them, but also which ones they themselves prefer, prioritise, or pay attention to. In previous studies, wayfinding preferences regarding cues and strategies have been investigated via focus-group interviews, qualitative interviews with individual VIPs, and surveys. Some studies focused on cues associated with one specific sensory modality, i.e., touch [22,38], smell [39], or hearing [17,40]. Other studies asked VIPs which points of reference are important to them and reported whether they perceive them through their sense of touch, smell, or hearing [41,42,43,44,45]. Cues that were reported in these studies include buildings (e.g., churches, schools, banks), shops (e.g., the smell of bookshops, flower shops, shoe shops), and food establishments (e.g., the smell of restaurants, bakeries, supermarkets, coffee bars), as well as particular features of buildings such as walls (in case of cane use), or entrances (e.g., hearing doors opening, or encountering stairs or sunken entrances with a cane). Furthermore, natural environmental cues were reported (especially the smell, sound, or feeling of trees, but also, e.g., the sound and smell of the sea), as well as human activity (e.g., hearing the sound of footsteps, crowds, children playing, or people waiting at a bus stop). Another well-represented category of cues in these studies is traffic-related, especially the sounds of vehicles (e.g., car horns, vehicles passing, vehicles braking, trams in the distance, identifying intersections, and discriminating between different types of vehicles).

Even though these studies have provided relevant insights on which sensory cues are experienced as helpful to VIPs, it remains unclear how these preferences and strategies regarding sensory cue use might vary between individuals or contexts. For instance, there is some evidence that these preferences depend on variable circumstances such as weather conditions, time of day, or how stimulus-rich an environment is [21,46]. Furthermore, although certain sensory cues might be experienced as helpful to the majority of the VIP population, it is possible that some VIPs prefer to rely mostly on their sense of touch, whereas others might prefer their sense of hearing. As far as we know, there is only one study that has aimed to systematically map those individual differences in subjective navigational preferences, albeit in the context of formulating personas and improving wayfinding technologies. Williams et al. [24] conducted qualitative interviews on several aspects of VIPs’ navigational experiences. They found two major themes within their data and formulated ‘types of navigators’ based on these themes. The first theme encompassed “Personality” and described how different personality characteristics varied between and were relevant in the way VIPs described their wayfinding experiences (i.e., Attitude towards exploration, Asking for help, Reliance on technology, Technology adoption, and Preferred mobility aid). The second theme, “Scenario,” described characteristics of the environment (i.e., Terrain, Familiarity, Weather, Crowd density, Transportation availability, and GPS availability). The types of navigators were formulated as VIP personas that include a short description of the individual’s wayfinding preferences and strategies focused on technology use, as well as four personal characteristics (e.g., which type of mobility aid they use). Also, in the context of improving technologies and inspired by the persona approach of the Williams et al. paper, Gupta et al. [19] characterized the wayfinding preferences of people with different kinds of impairments (including visual) through qualitative interviews. They concluded that the dimensions in which these preferences can vary are Technology (familiar vs. latest), Route (planned vs. spontaneous), Assistance (human vs. technological), and Experience (enjoyable vs. efficient).

Previous research offers important but incomplete insights into VIPs’ wayfinding experiences. First, studies that examined the use of technological cues (e.g., Williams et al.’s study [24]) might be dated, given the rapid development of navigation apps and assistive technologies in the past decade [47,48,49,50]. Second, studies that focus on specific sensory cues (e.g., Koutsoklenis and Papadopoulos’s array of studies [38,39,40]) identify which cues are reported but provide limited understanding of when and why these cues are preferred in practice. Crucially, the usefulness of sensory cues is not fixed but depends on circumstances and personal context, an area where systematic evidence and comprehensive overviews remain relatively scarce. Third, broader reviews (e.g., Park et al.’s work [21]) do outline personal and environmental factors relevant to wayfinding across disabilities, but do not provide an in-depth description tailored to visual impairments or link these contextual factors to cue preferences. The present study addresses these gaps by offering a novel, in-depth, up-to-date classification of multiple types of cues by directly looking into VIPs’ motivations, considerations, and reflections on which, when, and how to use different types of cues during wayfinding. In this way, we integrate these cue preferences with personal and environmental factors, offering a level of synthesis not previously available.

The goal of the current study was therefore to investigate navigational preferences of VIPs, particularly in terms of their preferred sensory modalities, as well as mapping which personal and situational factors are relevant for these preferences. To map out these subjective navigational preferences, semi-structured qualitative interviews were held with VIPs, asking them to describe a well-known route in their daily lives. Thematic analysis was used to systematically interpret and classify VIPs’ wayfinding experiences. From our thematic analysis, we conclude which informational resources VIPs use during navigation and how (i.e., sensory modalities, existing spatial knowledge, assistance from others, and smartphones), and which factors influence resource choice and strategy choice (i.e., navigational intentions, affective factors, and environmental factors impacting how sensory information is perceived).

## 2. Materials and Methods

### 2.1. Sample

We interviewed eleven people with visual impairments (VIPs). The sample consisted of four women and seven men, ages ranging from 33 to 73 years (mean age 53 ± 11 years old). Based on self-reported symptom descriptions, three participants had late-onset visual impairments (i.e., diagnosed at age 16 or older), one had early-onset impairment, and six had a congenital impairment. Furthermore, we categorized three participants as totally blind, three as very severely blind, two as severely blind, and three as having a moderate visual impairment (in line with [51]). Six of the eleven participants indicated that they currently have or have had a guide dog in the past, and nine people indicated making use of a white cane (symbol canes, pencil tip canes, and ball tip canes). Participant 01, 02, 04, 05, 08, 09, and 10 have participated in O&M training (ranging from about one to three times in total throughout their lifetime, for about 10 times up to two years of training per trajectory, where single sessions lasted for one or two hours). Participants 07 and 11 did not follow the O&M training to learn routes. From participants 03 and 06, we did not receive information concerning their prior experience with O&M training. All interviews were conducted in Dutch, as all participants were natively Dutch and lived across the Netherlands. Individuals were excluded from participation if their visual impairment resulted from a neurological disorder rather than an ocular condition, and/or if they reported sensory issues such as (partial) hearing loss in addition to their visual impairment. An overview of the sample characteristics can be found in Table 1. A brief case summary of each participant can be found in Appendix B. In these case summaries, the daily activities of the participants are described, as well as the main themes that were most important to this participant.

Participants were recruited via The Eye Association Netherlands and Bartiméus. Participants recruited via The Eye Association or via Bartiméus received a gift card of EUR 15 or EUR 8, respectively, for their participation. All participants received reimbursement of travel costs when applicable. Informed consent and data management procedures were carried out according to a protocol approved by the Ethics Review Board of the Faculty of Social & Behavioural Sciences of Utrecht University.

### 2.2. Procedure

**Figure 1:**
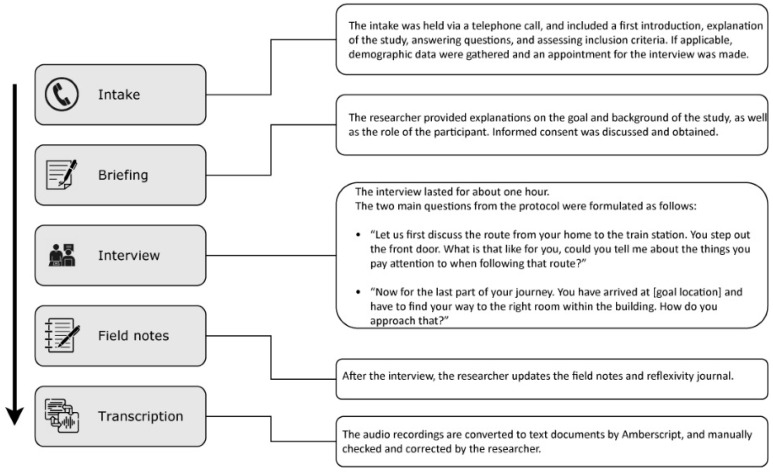
Interview procedure. The steps taken per interview per participant.

Face-to-face, semi-structured, qualitative interviews were conducted with the participants. First, an intake conversation was held via a telephone call with each interested participant. The goals of this conversation were to inform the interested participant, to check whether the interested participant met the inclusion criteria, and to make an appointment for conducting the interview when applicable. Each appointment lasted for about one and a half to two hours. The actual interviews lasted approximately one hour (ranging from 54 min to one hour and 27 min). An appointment started with reiterating the goal and the background of the study. During this explanation, the participant was given multiple opportunities to ask questions. Thereafter, the informed consent form was discussed and written consent (or oral consent, in case a participant was not able to write) was obtained, after which the actual interview would start (see Figure 1). The audio of each interview was recorded on two separate audio recorders (Olympus WS-853, and Olympus WS-852 (Olympus Corporation, Hamburg, Germany) as a backup). After the interview, the participant was thanked for their participation, asked about their experience of being interviewed, and briefed about the next steps of the study. After each interview, the researcher’s field notes and reflexivity journal were updated [52,53]. The goal of the field notes and reflexivity journal was to monitor how the study design, analysis, and results could have been influenced by the research question (e.g., choice of questions during the design of the interview protocol; see Section 2.3), theoretical concepts and literature (e.g., coding formulations during analysis; see Section 2.4.3), possible power dynamics due to the sightedness or assumptions of the researcher and the visual impairment of the participant (informing, for example, participant instructions and question formulation; see Appendix A), or the researchers’ subjectivity (e.g., clarifying through discussions within the research team whether concerning which findings sparked curiosity or surprise, for whom, and why).

### 2.3. Design of the Interview Protocol

The interview protocol was designed to follow the structure of introductory questions, transition questions, key questions, and closing questions [52,54,55]. The key questions were constructed in such a way that a participant would describe a familiar route in their daily lives and the cues they encounter along that route, in which each protocol question concerned one part of the route. Most participants chose a route they travel weekly (though the so-called first-mile could be travelled on a near-daily basis), or 1–3 times a month. One participant chose a route they travelled every 2–3 months. The routes were systematically discussed, first discussing navigation inside one’s own home towards their front door, then traveling outdoors towards a public transport stop, navigating inside public transport vehicles, and navigating outdoors towards the chosen destination, and lastly, navigating indoors at the destination to the exact room or space. The key questions were formulated as “First, the journey to [the bus stop]. You step outside. Can you tell me what that is like for you, which things catch your attention when you navigate there?” and “Then you arrive at your destination. Now you need to find the exact room in the building. How do you do that?”. What-questions were included to be able to extract the types of cues that are important to the participant and how-questions were included to be able to extract strategies from the participant’s responses. That way, the narratives of the participants could be used to extract all relevant information to answer our research questions.

The protocol included likely follow-up questions to the key questions. The purpose of the follow-up questions was to inquire further about which types of cues or information the person encounters and what those mean for them, and thus fill relevant gaps in the person’s route descriptions. Exemplary follow-up questions are “how do you know that landmark is there at [specific point in the just described part of the route]?”, “can you tell me more about the things you hear during [the just described part of the route]?”, or “how do you know that you have to go left at [specific point in the just described part of the route]?”.

After designing and constructing the protocol, the structure of the protocol and the formulation of the protocol questions were assessed by the research team. Last, a pilot interview was conducted with an expert by experience (i.e., a visually impaired researcher with experiential expertise). The final (translated) interview protocol can be found in Appendix A.

### 2.4. Data Analysis

#### 2.4.1. Pre-Processing

The raw data consisted of the audio recordings of the interviews. Each audio recording was converted to a text document using speech-to-text software [56]. Each transcript was checked and corrected manually by the first author (DB) according to an intelligent verbatim approach. In this correction phase, identifiable information was anonymized. The corrected transcripts were used for the data analysis.

#### 2.4.2. Thematic Analysis

**Figure 2:**
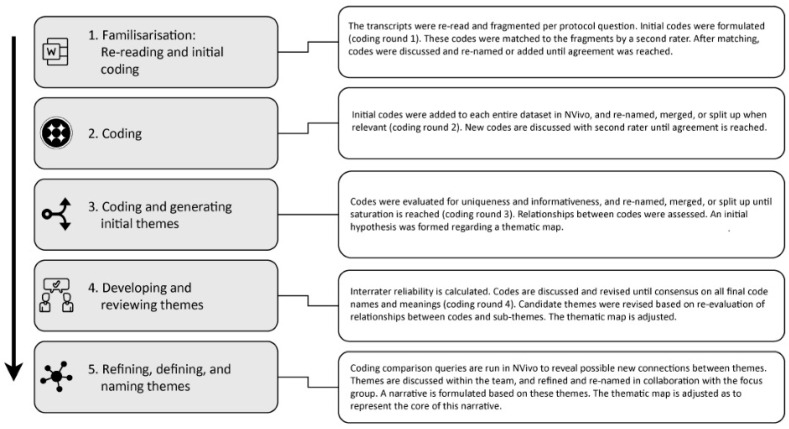
Thematic analysis. The thematic analysis of the interview data consisted of coding (four rounds), formulating themes, and constructing a thematic map. The figure shows the five steps in which this was achieved.

Thematic analysis of the data followed the steps as outlined by Braun & Clarke [57]. That is, Familiarising yourself with the dataset, Coding, Generating initial themes, Developing and reviewing themes, Refining, defining and naming themes, and Writing up (see Figure 2). Coding was done by the first author (DB) in Microsoft Word and NVivo (v14.23.1, [58]). Inductive coding was used during the coding phase to interpret the data (i.e., fragmenting the text, providing each fragment with one or multiple codes, and theme development were directed by and reflect the literal content of the data, as opposed to starting the analysis using an existing list of possible codes; [52]). In total, four coding rounds were carried out. In coding rounds one, two, and three, co-authors KO, NS, or AP functioned as a second rater. Codes were compared, discussed, and altered based on insights during those discussion sessions until verbal consensus and saturation were reached. We determined saturation based on code meaning [59]. Saturation was considered reached when all data were captured by the generated code categories (i.e., (sub-)themes), under the assumption that data that could not be captured by the existing (sub-)themes would require additional data collection in order to find new (sub-)themes that do. All data relevant in context of our research aims were captured by the reported themes. We therefore judge that the dataset was sufficiently rich to capture the most salient themes and develop a coherent and meaningful thematic structure [60]. The fourth coding round started with a second rater (co-author ML) coding a sample of the data, which consisted of three out of 11 interviews (i.e., 10–25% of data units; see [61]). For these codes, the inter-rater reliability was calculated via the coding comparison query in NVivo [57,61,62]. For codes with an observed inter-rater reliability lower than a Cohen’s kappa coefficient of 0.4, the first rater (DB) and the second rater (ML) would repeatedly discuss and revise the code names and code descriptions until any confusions were solved, after which the new codes were coded again by the first and second rater. This process was repeated until a Cohen’s kappa coefficient of 0.4 or higher was reached.

To support the coding process and the formulation of themes, multiple coding comparison queries were carried out in NVivo to reveal possible meaningful connections between codes or themes. First, coding comparison queries were carried out to find out how often certain codes co-appeared (e.g., different types of (sensory) cues in different contexts or with different types of wayfinding aids). Secondly, coding comparison queries were carried out to find out how often certain subsets of codes were mentioned by each participant (e.g., concerning orientation and mobility, but also concerning specific cue use). A coding diary was kept throughout the coding process. Figure 2 shows a flowchart of the steps taken for the thematic analysis.

#### 2.4.3. Theme Development and Thematic Map Design

The thematic map is the main outcome of the study. It was construed based on data-driven decisions. The main factors in its construction were how often our participants brought up certain topics, why participants brought up these topics, and which topics were often discussed in the same text fragment. These factors were used to determine which codes to include in the map, and which associations between codes or (sub-)themes to add. Additionally, the map construction was informed by existing literature and the research design. First, the existing literature helped in formulating code names and (sub-)theme names. An example of this is the formulation and distinction between the orientation and mobility sub-themes (see, e.g., [1,2]). Second, design-driven considerations were also used to formulate (sub-)themes in the sense that the interview protocol was specifically focused on certain concepts. For example, the distinction between indoor and outdoor wayfinding was specifically included. Furthermore, some of the distinctions between codes could also be based on the research design, as our inquiry was focused on differences between cues from different sensory modalities.

Regarding the structure of the thematic map, a main theme can serve as an overarching theme for multiple related sub-themes. A higher number of sub-themes implies that our participants talked about this main theme in a more diverse way compared to themes with a lower number of sub-themes. However, this does not necessarily mean that participants talk about the theme more often. In other words, if a theme has three sub-themes, then people talk about the theme itself, but there are three distinct topics that are related to this theme that they also discussed in a way that is relevant to navigation.

#### 2.4.4. Focus Group

We consulted a focus group consisting of five members. Three of them are orientation and mobility instructors (one representative for each of the three collaborating organisations), and two of them are experts by experience (that is, people with visual impairments who are also experienced researchers). Before the start of the data collection, one of the experts with experience participated in the piloting of the interview protocol. After the thematic analysis, the concept map was presented to the focus group. First, a text document was sent to the focus group as preparation for a meeting. During the meeting, the thematic map was presented to the focus group members. They were asked to comment on these results, ask clarifying questions, and share their own experiences with regard to the codes displayed. The main input from the focus group that shaped the results were that the focus group members showed recognition with regard to the themes, that none of the members pointed out any themes that seemed to be missing from their perspective, a discussion on the difference between the ‘Mobility’ and ‘Safety’ codes, and a discussion on the placement of the ‘Energy and concentration’ code. Specifically, the discussion on the difference between the ‘Mobility’ and ‘Safety’ codes resulted in a more detailed code description of these two codes. From this clearer distinction, it appeared that data labelled with the ‘Safety’ code almost always involved mobility-related issues, but data labelled with the ‘Mobility’ code did not necessarily involve safety-related issues. This provided additional justification for keeping two distinct codes instead of merging them. The discussion on the placement of the ‘Energy and concentration’ code within the structure of the thematic map suggested an all-encompassing influence of energy management on wayfinding preferences, which was consequently further analysed.

## 3. Results

### 3.1. Theme Overview

**Figure 3:**
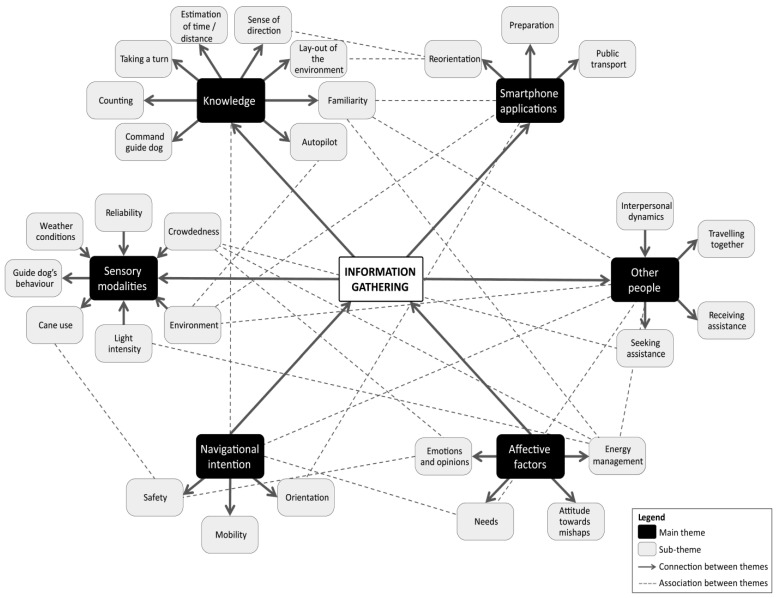
Thematic map. This figure shows the narrative on information gathering during navigating while visually impaired (in the middle), which is constructed on the basis of six main themes (in black), each with three to eight concomitant sub-themes (in grey). Grey arrows represent connections within a theme. An inward arrow indicates a smaller theme being a manifestation of the higher-order theme, and an outward arrow indicates an influence of a lower-order theme on the higher-order theme. Grey dotted lines represent associations between themes (i.e., themes that are discussed simultaneously in ways relevant to information gathering strategies).

The thematic analysis resulted in a network of six main themes, each reflecting patterns in what participants emphasized during discussions about navigation—both in terms of frequency and depth. These themes were developed to address three core aspects of navigational behaviour: (1) the types of information or cues people with visual impairments (i.e., VIPs) seek during navigation, (2) the strategies they use to obtain and apply this information, and (3) the reasons for choosing these cues or strategies (i.e., factors that influence the use of specific cues and strategies). The six themes identified are Sensory modalities, Knowledge, Other people, Smartphone applications, Affective factors, and Navigational intention. The first four themes (i.e., Sensory modalities, Knowledge, Other people, and Smartphone applications) capture the three core aspects of navigational behaviour: types of information, strategies, and influencing factors. In contrast, Affective factors and Navigational intention primarily describe the underlying motivations and contextual influences shaping cue and strategy selection. The themes and their relations are summarized in a thematic map (Figure 3).

### 3.2. Wayfinding Cues

**Figure 4:**
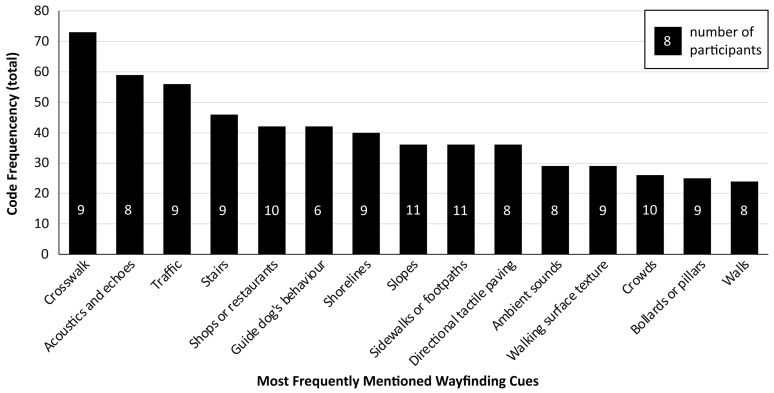
Cues relevant for navigation. This figure shows the fifteen most frequently mentioned navigation cues in the entire dataset. The height of the black bars represents the total number of mentions reported. This includes multiple mentions by the same individual(s). The number inside the black bar indicates by how many individuals a cue was mentioned (max. 11). Note that the cue Guide dog’s behaviour was mentioned by all six participants who own or have owned a guide dog.

Before looking further into the strategies used to obtain navigational information, the information deemed relevant by participants for reaching their destinations is reported first. In Figure 4, the 15 cues are shown that were reported most frequently in the context of navigation (i.e., cues that were mentioned over 20 times in the total dataset). Although these navigation cues were mentioned most often, they were relevant to our participants for varying reasons (e.g., depending on the context in which a person is navigating, the goal for which the information is meant to be used, or certain needs or priorities).

### 3.3. Sensory Modalities

Having established which types of cues are relevant to our participants, we now turn to the main themes we found that describe the strategies employed to obtain this information, and the factors influencing cue use. The choice for adding Sensory modalities as a main theme was predominantly design-driven. This was done because the main research question (as motivated in the Introduction) concerned the reported uses of hearing, touch, and a combination of the two as a means of gathering relevant information during wayfinding. This main theme, therefore, also informed how the interview protocol was designed (e.g., participants were asked to share how they use their sense of hearing to gather information during route-following). Participants appear to be using all their senses for navigation (i.e., hearing, residual vision when possible, touch, olfaction, vestibular/proprioceptive cues), as well as gather useful sensory information using their cane and guide dog (discussed below in the sub-themes Cane use and Guide dog’s behaviour). Besides discussing which sensory cues VIPs use and why, VIPs also discussed the circumstances influencing when, how, or whether to use these cues. Below, these findings are divided into several sub-themes: Environment, Weather conditions, Light intensity, Crowdedness, and Reliability.

The sensory modalities that our participants mentioned most frequently are hearing, residual vision, and touch (214, 102, and 95 times in total, respectively), but olfaction and vestibular/proprioceptive cues were also mentioned (48 and 20 times, respectively). Most participants reported using multiple sensory cues to perceive different objects at a certain time and location, rather than integrating information from multiple sensory modalities to perceive a single object. The primary context in which the strategy of combining multiple different cues was employed was during moments of doubt. That is, VIPs mentioned having a main cue to orient themselves, and shifting their attention to a cue from either the same or a different sensory modality as a check. Another reason for purposefully combining cues is that one of the cues might disappear at some point in time. When this would happen, it would still be possible to orient oneself using the remaining navigation cue(s).

Sometimes, participants would indicate having an explicit preference, or giving priority to one strategy or sensory modality over another, given the circumstances. Notable findings include variations in walking speed preference, with some individuals favouring a faster pace while others prefer a slower one. Additionally, preferences for sensory cues vary in busy environments. Namely, some participants prefer focusing on their hearing, as some sounds will stand out to them amongst the buzzing of the surrounding noise. In contrast, other participants report they prefer using their sense of touch, as they find it more reliable than using their hearing.

#### 3.3.1. Hearing

Our data suggest that the auditory modality is experienced as the most useful sensory modality for navigation (it was the most frequently mentioned). Specifically, VIPs report using local and distal sounds, acoustics, and echolocation to know where they are and know where they have to go when going from A to B. One participant explained how they orient themselves when going for a leisurely walk on the heath:

“*Well, then it’s just- and I like taking the time for that, and then I walk, and then I orient myself on the sounds I hear. Yeah, for example the train or children playing, because you know ‘hey, I am close to a playground here and that is on such and such side of the village’. And then I know, I know kind of where I am.*”*—pp02*

Different types of sounds are used in different ways. Some sounds enter awareness passively, in the sense that they are not actively sought out, but enter awareness by encountering it. Examples of this include noticing a sound along the route, a change in acoustics (tunnels, walls, tree-lined paths, overhangs, indoor/outdoor open spaces, foliage, et cetera), or a change in the walking surface texture that is observed through the sounds resulting from cane use. Other sounds, in contrast, are expected to be encountered and are, as such, actively sought out. Examples include using one’s cane to identify objects based on the sound it makes when the cane hits them, which can either function as an expected reference point or as an expected obstacle to be avoided. Participants also use the echoes of their voice, footsteps, or sounds of their cane to identify surrounding objects (e.g., foliage or parked cars) or structures (e.g., bus stops or buildings). The perception of echoes and acoustics is often described as a feeling (e.g., a ‘dark feeling’ when transitioning from an open space to a tree-lined path, or a ‘high feeling’ when walking past a high- or mid-rise building). Furthermore, many participants depend on the broadcaster in public transport to know when they have reached their stop. Finally, traffic sounds are often discussed. First, the sound of traffic is used to recognize a street, an intersection, or a turn. Second, hearing the direction of the traffic is used to keep to a path. Third, traffic sounds in the distance are used to determine the direction to walk towards or away from. Other examples of sounds and acoustics that are mentioned as points of reference to walk towards include: walking towards an open space, walking towards a counter when hearing the voices of the receptionists, walking towards the beeps from the check-in/check-out gates at train stations, or walking towards the audible crosswalk signal to know where to cross the street. One participant had a noteworthy cue that they used to walk towards when wanting to reach their home. This represents the creativity that can be involved when navigating while visually impaired:

“*And I also have a very good navigation: the neighbours’ parrot. No, that’s really- I’m serious! When I just lived here, yeah, that sounds a bit stupid, but that is really- really fun and handy. It is fun and functional. That, that, that parrot will whistle like [makes bird sounds], then I already know like ‘okay, I think I’m getting close to the stairs now’.*”*—pp03*

#### 3.3.2. Touch

Our data suggest that VIPs find haptic cues to be useful for both orientation and mobility purposes. Regarding orientation, VIPs report feeling Braille signs at stair railings, elevators, or poles to check whether they are at the right train track or bus stop. Furthermore, a change in the type of walking surface that is felt with the feet is also used as a cue for recognising a point along the route. Lastly, temperature changes are experienced as useful. For example, one participant remarked that they feel the heat coming from within a store and use it to recognise where the store’s entrance is. Regarding mobility, VIPs report using their sense of touch for avoiding obstacles and noticing steps up or down. Pp05 gave an example of trying to avoid bumping into low objects (planters, in this case):

“*I happen to know that they’re there. Yeah, but yeah, my dog will go around it of course, but if I could feel it with my cane I would- at some point I would just stick my hand out and try to feel if I could walk around it.*”*—pp05*

Sometimes, the same haptic cue is useful for orientation and mobility purposes at the same time. This is the case when, e.g., using a wall, fence, or gutter for following a path or walking in a straight line (i.e., mobility), as well as progressing on the route (i.e., orientation). Moreover, the end of such a shoreline is also used by VIPs as a cue to know where they are on the route or where to take a turn (i.e., orientation).

#### 3.3.3. Residual Vision

Vision is still experienced as an informative source of information by participants who have residual vision. The more they have, the more they tend to focus on visual information during navigation. In our sample, pp07, pp09 and pp11 are the VIPs with the largest amount of residual vision left (respectively 12–15% in both eyes, 10–12% residual vision in the left eye, and 10% residual vision in both eyes). To illustrate, pp07 is able to see the sidewalk next to a cycling path when cycling and uses it as a guiding line. She virtually never spoke of using her sense of hearing or touch during route-following. When she did, it seemed that it was more of a distraction to her than useful information. Likewise, pp09 often responded that he uses his vision when asked how he recognises a certain landmark or turn. Lastly, pp11 indicated that sounds are only really useful to him when he is trying to safely cross a road, as his hearing helps him in identifying any approaching vehicles. Nonetheless, even for other participants, whose residual vision was nearly non-existent, the minimal visual information that they did obtain, such as movement, light-dark contrasts (in the physical environment as well as on a smartphone screen), or bright colours, was still found to be useful. To illustrate, one participant describes the role their vision plays in how they find their flat’s front door when leaving the building:

“*And then, yeah, you walk straight on until yeah, I try to immediately think and kind of visualise the route a bit. But until you arrive at a doormat and with a door that opens automatically, you hear it open, so that, that helps, and if I’m lucky, I see something like, like light, that comes from outside. When it’s light outside at least.*”*—pp08*

#### 3.3.4. Olfaction

Olfaction is mostly mentioned as being used to identify public spaces like shops or restaurants. Common examples include supermarkets, cafés, soap shops, cheese stores, and bakeries. The smell of a particular shop can serve as a landmark, as well as a confirmation that one has arrived at a certain target location. Besides shops or restaurants, other smells that are mentioned are associated with nature. For example, the smell of birdseed in an aviary, the smell of grass, and the smell of horses in a meadow. The following example illustrates using one’s sense of smell explicitly in shopping areas:

“*I think, if you walk more in a, in a village or an actual shopping street, you do a lot more with your sense of smell. Also what I just said. Like, you know where a butcher is, so you know, like, I’m in the right place. You know where the [well-known Dutch variety store] is, you know where a, a, a drugstore, that you do a lot more with your, with your sense of smell and less with your sense of touch. I think, I know that for sure, yes.*”*—pp04*

#### 3.3.5. Vestibular and Proprioceptive Cues

Participants mention noticing speed alterations as well as bends or bumps in the road when traveling in a vehicle in order to orient themselves. Pp05, e.g., would know when the bus arrives at a certain station based on feeling the bus turn at roundabouts, besides hearing the train station in the distance, and noticing when many passengers would leave or enter the bus. Furthermore, participants notice slopes when walking, which are also used to orient oneself:

“*Another example: when I take my waste to the container, that container is about 50 m away. Now I can start counting the steps, but almost at the corner the street is a bit elevated. I feel it in my legs, it elevates a bit and then I know ‘oh, now I’m at the corner’.*”*—pp01*

#### 3.3.6. Cane Use

Most cane-using participants use their cane to progress along a route by following a continuous cue. Namely, they mention the following shorelines, such as walls, fences, and grass-sidewalk boundaries, as well as using the end of a shoreline as a useful cue (e.g., for judging when to take a turn, or where the entrance of a building is). In contrast, VIPs with guide dogs tend not to use their cane for following continuous cues, but rather as a check when arriving at a certain point. Examples of this are feeling a reception desk indoors, feeling the steps at a staircase or train entrance, or tapping against a fence to hear the sound it makes as a check. Tapping the cane can also make a sound that tells the navigator something about the height of an object, which can be useful when trying to identify or avoid obstacles. Furthermore, sounds as a result of cane use can also give information about the acoustics of a space. This information could be useful to check whether they have arrived at a staircase (which can make a more hollow sound), or to discriminate between a wall and an open space. Lastly, the cane is a tool to identify transitions in walking surface texture, which can be used for orientation. Feeling a change in walking surface with the cane is often mentioned in the same passage as hearing this change as well, especially with a ball tip cane. Lastly, the cane is also used for safety purposes, primarily for recognizing unevenness in the walking surface or managing steps up or down:

“*I mainly also use my cane for that if I indeed have to cross the street. And of course there’s a step down and a step up, you know? Step down at the sidewalk, cross the street, step up at the sidewalk. I definitely use my cane well for that, because that height, those heights can vary quite a bit. Right?*”*—pp09*

Some VIPs explicitly prefer a cane over a guide dog, since a cane provides more information about the environment or increases their sense of freedom. Negative aspects of cane use are that the cane can get stuck or caught, that canes are of no use when it comes to navigating a Shared Space (i.e., no discriminable features on the ground), and that travelling autonomously with a cane can require a lot of time and energy.

#### 3.3.7. Guide Dog

Regarding orientation, participants who walk with a guide dog explained that they can gauge their location on a route based on their dog’s behaviour:

“*Then I pay attention to my feet, to paths, how I walk, with my cane. That I swing my cane back and forth, that I can also feel ‘oh yeah, there, that’s about where I have to go left’. And that’s also a nice one: on the path where I walk into the forest, halfway I know that my dog always goes for a drink. Always.*”*—pp05*

Positive aspects of having a guide dog are that dogs are especially useful for finding fine-grained navigational goals both indoors and outdoors (mentioned most frequently), that it gives the navigator a reason to go outside, that navigating is faster and more relaxed than navigating with a cane only, that it is fun, and that it provides a sense of freedom in terms of mobility. Negative aspects of guide dogs include that dogs can also get lost and consequently lead their owners in the wrong direction (mentioned most frequently), that they can get distracted (e.g., by food on the floor), that there can be miscommunication between the navigator and their dog, and that training them takes a lot of time. Neutral aspects of guide dogs that are mentioned are that you need more space when navigating with a dog (e.g., wider paths, and more spacious seats in public transport), that dogs need to be cared for, that a dog is not useful for distal navigational goals or target locations, and that there needs to be a certain level of trust between the navigator and their dog. For one of the participants, the level of trust between dog and VIP affects the VIP’s willingness to travel longer routes or to unfamiliar environments. Lastly, walking with a guide dog allows the navigator to pay less attention themselves to certain aspects of the environment, which was mentioned as a positive aspect by dog users and as a negative aspect by cane users without dogs.

#### 3.3.8. Environment

The data suggest that the type of environment significantly affects participants’ navigational experiences, with notable differences between indoor and outdoor settings, and between open and cluttered spaces.

Indoor/Outdoor. Even though the interview protocol questions concerned both indoor and outdoor spaces, the discussions on indoor navigation were considerably shorter than on outdoor navigation. Of course, the actual routes indoors are generally shorter than routes outdoors, so there is less to discuss. But VIPs also mention that—rather than consciously searching for cue after cue like they do outdoors—an indoor environment is either so familiar to them that they move through it on autopilot based on their existing knowledge of the space (e.g., the layout of a floor, including where the windows and door openings are), or that an environment is so difficult to navigate autonomously that they report just asking someone to walk them to the desired space. Problems that participants encounter indoors are usually related to a lack of useful shorelines and not being able to find fine-grained navigational goals (e.g., finding a seat in a theatre, finding a ticket machine at the town hall, or finding a product in a store when the store’s layout has changed). Asking for help was the most common way to gather spatial information indoors compared to using one’s senses or existing spatial knowledge.

Open spaces. Another aspect of a type of environment that came up was open spaces, such as squares. Cane users without a guide dog experience problems with large, uniform public spaces. Particularly, pp04 mentioned being bothered by Shared Spaces, because they lack shorelines. Also, pp03 talked about the risk and fear of losing their orientation and subsequently wandering around at nighttime, getting lost in a schoolyard, unable to find the exit. Participants reported preferring to avoid routes through open spaces when possible.

#### 3.3.9. Weather Conditions

Weather conditions are perceived as either advantageous or disadvantageous for certain navigational goals. For example, participants report that snow makes it harder to feel steps up and down or see the contrast between the street and the sidewalk. Another example would be feeling the wind at a certain point along the route to recognize an alley, or using the warmth of the sun on one’s face to estimate cardinal directions. An additional example is that the reflection of the bright sun on a road on sunny days is reported to make it harder to identify other road users. Lastly, there are some contextual aspects relating to weather conditions that were relevant to our participants. Some participants report that, for example, the noise of the rain or hail can be overstimulating, which impacts their focus. Other participants reported having unpleasant experiences relating to weather conditions, such as pp05, who told a story about suddenly feeling a bunch of wet tree leaves on their face that were hanging lower than usual due to the added weight of the leaves after rainfall, and how their dog was not able to account for higher obstacles.

#### 3.3.10. Light Intensity

Navigating in daytime versus nighttime sparked different discussions amongst our sample. Navigating during the daytime is experienced as easier for participants who still have some residual vision that they rely on. However, our participants do report being bothered by bright sunlight, which can impact the navigator’s energy levels. Navigating during nighttime seems to be more challenging, as there are fewer people outside who you could ask for help when needed. Moreover, bright streetlamps or headlights are also experienced as bothersome when it is dark outside. Finally, with regard to indoor navigation, the lighting at some stores can be too bright to be able to comfortably navigate comfortably. It can also be a struggle to deal with the transition from an outdoor environment to an indoor environment because of the change in light intensity.

#### 3.3.11. Crowdedness

The number of people and human activity in an environment seems to affect our participants’ wayfinding experiences. Some of our participants prefer busy environments over quiet environments, though others vice versa. On the one hand, busy environments can be anxiety-inducing or experienced as difficult because it increases the chance of collisions. Busy environments can also be experienced as rather draining in terms of energy management, as the noise can make it harder for someone to filter the sounds to orient themselves properly compared to quiet environments. Although quiet areas are not as energy-consuming as more crowded areas, a reported disadvantage of quieter areas is that there are fewer opportunities to ask for help when needed. Another reason for preferring busy environments over quiet environments is that more sounds are available to orient oneself, amongst which are those resulting from traffic or the behaviour of other travellers. For example, the sound of a crowd of people is said to be used as a beacon, or the direction in which the crowd is moving is used to follow along towards the exit. Moreover, determining the walking direction of a crowd based on sound and sight is also helpful to orient oneself, e.g., in train stations. Lastly, the sound of groups of children playing is also used for orientation, for example, to identify a school that one passes on route that can serve as a landmark.

#### 3.3.12. Reliability

The reliability sub-theme refers to the extent to which cues are conditionally present. Such cues are helpful for orientation when experienced along a certain route, but they are not always perceivable. This applies to all kinds of sensory cues that are mentioned above. That is, some cues are bound to a season (such as smelling the blossom trees), bound to weather conditions (such as feeling the warmth of the sun), or bound to a time of day (such as the smell and sound of certain catering facilities). Furthermore, one participant (pp08) noted that travelling during the lockdowns due to COVID-19 was challenging, as the escalators were turned off at the train station. As a result, they could not use the sound of those escalators anymore to orient. Other examples of unreliable cues are cues that depend on human-specific activities (such as bottle banks or pianos at train stations).

### 3.4. Knowledge

The main theme, Knowledge, represents all instances in which participants mentioned using existing knowledge for making navigational judgments or decisions. These instances include Autopilot, Layout of the environment, Taking a turn, Sense of direction, Command guide dog, Counting, Familiarity, and Estimation of time or direction.

#### 3.4.1. Autopilot

The Autopilot sub-theme represents a situation in which a person does not need to think or pay conscious attention to specific cues in order to go to a desired goal location. This might result from a combination of habit formation as well as existing knowledge of an environment, and being familiar with the environment’s specific quirks. Namely, participants mainly reported using autopilot in (very) familiar environments, both indoors and outdoors. One example of moving through indoor spaces on autopilot is navigating one’s office space:

“*I walk back kind of based on my sense of touch. I pick up my stick again and I walk back to the secretariat or do other things. And they say ‘how do you do that, because you can’t actually see anything?’. I say ‘well, I still have some residual vision, but I know exactly where I am when I take two steps from the kitchen’ and I don’t count them consciously anymore, but you just know where that table is. It’s so, very automatic.*”*—pp04*

#### 3.4.2. Layout of the Environment

Knowledge of the layout of an environment refers to the instances in which participants talk about the spatial structure of an environment, such as the relative spatial positions of multiple landmarks or roads, or specific aspects of a road, such as where it leads to, or the knowledge that it is placed next to a residential area. This refers to both indoor and outdoor environments. It also happens that VIPs without any (useful) residual vision left remember the layout of an environment based on visual spatial memory from a time they could still see something. Sometimes, participants merely talked about the layout of an environment without an apparent purpose. In some other instances, this knowledge is brought up with a link to a specific goal, such as taking a turn:

“*Or I hear a- Well, a very simple example: in our town [residential area], you have [street name 1] and [street name 2]. There is about 40 m between them, but they are connected to each other by means of an alley with a roof on both sides. And if you walk past and then a car happens to pass by in that other street, then you hear that and then you know ‘oh, this is where I have to go’.*”*—pp01*

#### 3.4.3. Taking a Turn

When participants report having to take a turn at points where multiple walking directions are possible, this is usually based on perceiving a specific sensory cue and using it as a directional cue. Alternatively, there are points along the routes at which the participants turn left or right based on knowing they should without using a particular sensory cue. Instances for which this is the case are knowing that you have to turn left or right directly after exiting an elevator, after ascending or descending stairways, or after exiting a bus, train, or tram.

#### 3.4.4. Familiarity

Our participants report dealing with familiar and unfamiliar environments in different ways. In the case of very familiar environments, participants report that it is easier to orient oneself when getting lost. Multiple participants told us their strategy for finding their way back after getting lost is to just walk around the area until coming across a landmark that they recognise. Unfamiliar environments are experienced as harder and more energy-consuming to navigate due to a lack of knowledge, not only in terms of orientation, but also in terms of mobility. In terms of orientation, multiple participants said that it is more difficult to find fine-grained navigational goals in unfamiliar environments. Pp11 told us how difficult navigating unfamiliar indoor environments is because the signage is different everywhere. Multiple participants indicated an explicit preference for navigating familiar spaces, and some participants prefer avoiding unfamiliar spaces altogether. In terms of mobility in unfamiliar spaces, encountering unexpected environmental features can be challenging, even when getting assistance:

“*And if you don’t know the way, then- but I did have assistance now, but yeah, then, you, you have a certain expectation pattern in your head, right? Often. And if you forget that there are those stupid three steps at [station], yeah, then it is often also, also, yeah, it does something to you, you know. Like yeah, yeah, it slurps energy again, so [laughs]. […] But that assistant ‘oh, there are some steps here’. But yeah, when she says ‘steps’, steps up, steps down? That’s quite a difference. Yeah, steps down are more risky than steps up.*”*—pp10*

*Doubt.* Participants report that doubt costs a lot of energy and that unfamiliar environments cause more doubt. Participants report having to constantly pay attention to what they are hearing and feeling to know where they are or reassure themselves that they are still going the right way. Furthermore, situations in which the explicit use of multiple sensory modalities was discussed were often in the context of doubt:

“*It’s just that when there is a truck in front, and I get a little bit of that smell, then you start to doubt, because that truck should never have been there in your experience. […] I didn’t hear any sound from the truck, and then you pass it and the smell of the cheese-nut store comes up. And then you go and check by feeling the gutter to see if you have the idea of the gutter, if you are on the right track.*”*—pp01*

*Unexpected changes.* Participants experience inconvenience from unexpected changes in the environment. This usually concerns construction in familiar environments, which (temporarily) changes the environment, including its landmarks and possible routes. An example of this is pp04, who told a story about feeling a sense of panic when getting lost on a very familiar route and feeling very puzzled as to how this could have happened. They then recognised that a distal cue was lacking that they subconsciously use to orient themselves. Namely, they realized that they normally use the faraway sounds of a highway that was unexpectedly under construction. Another participant mentioned missing their tram stop due to unexpected changes in a tram route familiar to them (pp11). An additional example is walking the same route, but unexpectedly encountering more obstacles because it is a garbage collection day (pp09).

#### 3.4.5. Sense of Direction

Although not often, a sense of direction was also reported to be of use for orientation during route-following. Sense of direction is particularly mentioned in the context of sensing a diversion from a correct route. This was mentioned by two out of three participants of our sample with the most residual vision, namely pp07 and pp11. One of these participants said, for example:

“*And yes, that is when I am not sure about the direction. That is it in particular. [thinks]. That I then just- [thinks]. And it can be because of the position of the sun or indeed the compass that I then go in a certain direction. I am- I think that I am always aware of the direction I am more or less walking in, or whether I am walking towards the north, or the west or the east. And I think, I think that I always have that in the back of my mind.*”*—pp11*

#### 3.4.6. Command Guide Dog

The sub-theme Command guide dog entails situations in which the navigator gives their guide dog a command to reach a particular navigational goal. This is usually a goal relating to orientation, such as finding a landmark or a fine-grained navigational goal, such as a post or pole, to navigate to from where the navigator knows they can safely cross a road. Participants note that giving commands to the dog is only useful when you are very familiar with a route or an environment, as you have to know when and where exactly to give certain commands. Moreover, the commands themselves also need to be sufficiently specific (e.g., ‘search door left’ is only effective when there is only one door on the left, or when the command is given at the exact right moment). The following piece of data represents a participant giving their guide dog a command in order to find their next beacon:

“*Yes, then you just walk quickly and then I say to my dog when I think I’m almost there, ‘find stairs’, of course you have a command for that too. And then when, when I know that there, he now walks kind of to the middle of the platform, I know we’re almost there. And then of course you use your cane again to feel “oh, here are stairs, right”. Treat for the dog.*”*—pp10*

#### 3.4.7. Counting

Counting is a strategy that is mentioned by every participant at least once. Participants mostly mentioned counting the number of stops while using public transport to know when to make the decision to get off the vehicle. Other situations include counting the number of steps to take, counting the number of houses to find a specific address, or counting the number of gutters to cross with a cane. Most often, though, VIPs specifically emphasized that counting is not their strategy of choice. Explanations given are that counting the number of steps to take is not possible for long paths, that counting doors when walking through a hallway involves the risk of hurting your fingers, and that counting the number of notches you feel with the cane takes up unnecessary space in their memory.

#### 3.4.8. Estimation of Time and Distance

Participants reported that an estimation of time or distance was useful information during wayfinding. Particularly, estimations of time and distance are related to the expectation of a certain cue at a certain time and place. In terms of distance, VIPs report distances of around 100–500 metres with regard to knowing a certain landmark will appear. In terms of time, VIPs report knowing how long their journey will still take at a certain cue or landmark. When the cue is not perceived, this can be a sign for the person that they have gotten off track:

“*I do a lot by ear. For example, if you are on a route, that you have to go past a kind of overhang and it takes forever and it takes forever before you come across the overhang. Then you think ‘shit, we had to go to that overhang, because then, I always heard that’, but then you are not, and then you know ‘hey, I am going the wrong way!’.*”*—pp02*

### 3.5. Other People

We found that other people are an important source of wayfinding information. Firstly, VIPs report using the assistance of other people in varying ways and in various circumstances. Secondly, VIPs talked about the social aspects of getting help, but also about all kinds of interpersonal dynamics of navigating in general. In sum, the Other people sub-theme is divided into Assistance and Interpersonal dynamics.

#### 3.5.1. Assistance

Our participants report being offered assistance either by people familiar to them, a passersby or a service employee. Passersby or service employees ask where they need to go and whether they need any help going somewhere, or give useful information such as whether a seat is empty or where a certain fine-grained navigational goal is. It is often appreciated by our participants when people offer their help in this manner.

Participants report seeking assistance in several instances. Many participants report asking for help during route-following or when being lost (e.g., asking passersby for directions to a certain landmark). Some participants ask for help when preparing for a journey (e.g., a loved one looking up the public transport transfers). Furthermore, participants ask for help with finding fine-grained navigational goals (e.g., finding a product in a store with the help of an employee). Participants also tend to ask for help as a check when they are feeling unsure (e.g., asking if they have found the right bus or train). Last, participants report walking along with an individual or a group of people as a strategy of getting to a desired goal location.

The strategy of walking along with others has three different forms. First, participants follow a crowd or an acquaintance when going for a stroll or for finding certain locations along a route using their sense of hearing (e.g., the click-clacking of heels), smell (e.g., the scent of a loved one), touch (e.g., holding someone by their arm or shoulder), and sight (e.g., a brightly coloured coat). Second, our participants report actively asking a passerby or service employee if they could walk them to a certain location along the route, so they can continue from that point on their own. Third, our participants report asking a contact person or loved one to pick them up so they can walk along for the last part of the route (i.e., from the final bus stop or train station to the correct building). The last part of the route, often unfamiliar, is experienced as the most difficult part:

“*But yeah, in [city], I really don’t know any, really no directions. Really not. Well, then I just ask ‘would you please-’, well, and then I’ll figure out ‘my train arrives at that platform at such and such a time’, say, ‘and I come with a, a white, with a white labrador’, like ‘can you come and pick me up?’. No, I’m not going to do that, then I’m just going to ask.*”*—pp05*

Our participants discussed several advantages and disadvantages of seeking assistance. Advantages are that it is faster than traveling autonomously (mentioned most frequently), that it saves energy, that it is useful for finding fine-grained navigational goals, and that it makes it easier to manage unexpected situations. Disadvantages are that the directions that passersby give are not always useful, that a service employee or passerby is not always present when you need them, that making use of certain services can be quite expensive, that it makes you feel dependent and it prevents you from building independency, that you can feel like a burden to others, and worries of being perceived as helpless.

Our participants report travelling together with friends, romantic partners, or colleagues to get to a certain location. In some cases, people specifically ask a loved one to travel together so they can drive them to a certain goal location. In most cases, however, the goal location was an event they were supposed to go to together anyway, such as a theatre club (pp03), a voluntary job (pp06), or a soccer match (pp08). Some participants tend to ask for help more frequently than others, for example, because they do not prioritize independence:

“*Because I myself also have a bit like, well, that’s nice, independent, you have to do that too, but sometimes it is also easy to, to let yourself be carried along because you, carried along, taken along, because it also saves energy that you can then use for your theatre in this case.*”*—pp03*

#### 3.5.2. Interpersonal Dynamics

Our sample reports various interpersonal dynamics that they encounter during their navigational ventures, both with strangers and with loved ones. Pp08 told stories about both positive and negative experiences. That is, it is funny when an equally visually impaired friend you are meeting up with can jump-scare you as a prank by suddenly touching you, but less so when a random sighted passerby is supposedly helping you by suddenly grabbing you and bringing you to the other side of the road, where you have totally lost your route and orientation. As pp06 succinctly puts it: getting lost happens at a square metre when you are visually impaired. Other examples of interpersonal dynamics include funny moments such as calling your brother, telling him you are lost, and him dryly replying that he can see you standing in front of the house (pp02), or getting comments from a loved one or stranger about, e.g., one’s tempo, courage, orientation skills (e.g., pp01, pp07, pp08).

However, there were also many worries related to the social context. For example, the worry of looking silly while searching for your landmark (pp08), worrying that you accidentally sit on top of someone when trying to find an empty seat in a train, tram, or bus (pp10), worrying that you are bothering others because of your tempo (pp07, pp11) or because your cane is tapping against people’s front doors when using the walls of the houses as a shoreline (pp01).

Moreover, interpersonal dynamics are involved when travelling together with loved ones, which can be both pleasant and useful (e.g., pp04, pp09). Pp01, for example, told a story about walking through a busy shopping street with their sister, to whom they enthusiastically explained using the word ‘hello!’ for echolocation purposes. Their sister then warned them that people would think they were saying ‘hello’ to them, after which they changed it to ‘wello!’. However, asking a loved one to drive or accompany you somewhere is sometimes difficult. PP03, for example, remarked that they make sure their loved one does not feel like a driver and that the activity they are going to will also be of interest to them.

Finally, participants report using social interactions specifically as a means to gather information. Sometimes, VIPs make small talk so they can follow someone based on the sound of their voice (e.g., pp01). In other instances, social interactions can be used to find fine-grained navigational goals. One participant, for instance, admitted that they purposefully make small talk in the waiting room at the hospital with a stranger who found the ticket machine right before them, so they can figure out where the ticket machine is and when to continue on their way:

“*And then I have found that ticket and then I still don’t know anything. Then I have to ask ‘what ticket number do you have?’, ‘I have number 90’, well, then I have number 91. But nothing else is said. So you keep a very close eye on that man. You’re gonna talk to him. And only when he is gone is it my turn.*”*—pp06*

### 3.6. Smartphone Applications

Although there were no specific questions in the interview dedicated to the use of navigation apps, participants did bring up using several different apps. They use apps for orientation during route-following, for planning a route during preparation, for checking schedules and updates in case of public transport, or for finding the correct route again after getting lost. Most VIPs mentioned only using an app in unfamiliar environments. However, not all the VIPs make use of apps. Notably, pp03, who was born without eyes, said that they wished they could use a navigation app, but they just cannot understand the instructions or use the app properly.

During route-following, smartphone applications are mostly used to receive different types of auditory-verbal instructions for orientation. Examples include receiving information on when to take a turn, where a certain landmark is, how far, and in which direction one needs to walk, using the absence of instructions as a sign of still being on the correct route, and receiving a sound cue when the goal location is reached. Navigation apps are also used to check one’s location or orientation in case of doubt. Furthermore, pp07 and pp11 use their smartphone to take pictures of signs, so they can zoom in on the photo and read the information on the sign.

Several different apps were mentioned. We found that our participants primarily use Google Maps (mentioned 74 times by 8 participants), as well as Nav by Via Opta (mentioned 38 times by 10 participants), Lazarillo (mentioned 10 times by 3 participants), and eZwayZ (mentioned 6 times by 2 participants). One participant (pp06) mentioned Captain Mobility (not supported anymore), komoot, Viktor Reader Trek, NaviLens, and Here WeGo. Other apps that were mentioned by one participant include Maps.me (pp11) and Apple Maps (pp10). There were also multiple people who talked enthusiastically about the usefulness of BlindSquare for gauging directions specifically (mentioned 12 times by 3 participants):

“*Which, especially in which direction I should walk. So the BlindSquare that, that also says ‘five past twelve’, right. That, that, that with the hands of the clock where you should go. I just really like that. Which, especially which direction you should walk. And how many meters it is. […] So in- the meters I find very important, which direction I should go. So with the hands of the clock of which direction you should walk. Whatever is around me, I have all those settings, I have a lot turned off.*”*—pp04*

Advantages and disadvantages of using smartphone applications were also discussed. Advantages of apps are that they provide updated information on current circumstances and that it increases independency. Disadvantages are that they are not precise enough in terms of localisation (mentioned most frequently), that instructions can be rather unclear, that using an app can cost a lot of energy, that the absence of instructions can spark some insecurity about whether one is still on the correct route, that navigation apps that are designed for VIPs can be rather expensive, that an app cannot be used for mobility (e.g., it does not indicate obstacles), and that app use is dependent on Internet access. With regard to the unclear instructions, VIPs differ in which types of information they would like to get (e.g., only getting unambiguous cues when to take a turn, getting a cue when they are approaching a zebra crossing, or receiving vibrations that would help them walk in a straight line). A need for smartphone apps is especially expressed in the context of indoor navigation.

#### 3.6.1. Preparation

A dedicated question in the interview protocol inquired how VIPs prepare for their journeys. Smartphone applications are used to prepare for a journey in relatively unfamiliar environments. For example, apps are used to check walking surface textures of the paths VIPs intend to take, to check the accessibility of certain public transport stops, or to check where the entrance of a building is.

#### 3.6.2. Public Transport

Our participants reported often using smartphone apps to check the status of their public transport journey both before and during their trip. Information that they tend to look for is whether the tram(s), bus(es), and/or train(s) were running on time, whether there are unexpected situations concerning their journey (e.g., constructions), transfers, and how many stops they have to travel. Applications that our VIPs use are the OV-info app, the 9292 app, and the NS Perronwijzer app or NS Reisplanner.

#### 3.6.3. Reorientation

Question 11 of the interview protocol concerned people’s experience regarding getting lost, including follow-up questions on recognizing being lost and strategies for finding the way back to the correct route (i.e., reorientation). To reorient themselves using their smartphone, VIPs select a familiar location along their usual route in a navigation app and let the app navigate them to that point to get back on track.

### 3.7. Affective Factors

One of the two main themes that influence cue use and strategy choice is named Affective factors. This theme concerns personally relevant factors that are mentioned by participants themselves in the context of their wayfinding habits. The patterns found within this main theme are formulated as Energy and concentration, Emotions and opinions, Needs, and Attitude towards mishaps.

#### 3.7.1. Energy and Concentration

Participants talked about situations that cost them a lot of energy, focus, or concentration, as well as situations in which they explicitly made navigational choices to manage their energy levels. For example, navigating in unfamiliar environments takes up a lot of energy because there is more doubt and because continuous monitoring of your whereabouts and safety is needed. Another aspect of this theme is talk of distractions (e.g., chatting with someone can be distracting when having to orient oneself, as well as having the radio on when driving, or experiencing the sight or sound of traffic as noise in a busy environment). Furthermore, using one’s sight can be experienced as energy-consuming. For example, pp05 deliberately chooses not to focus on their sight for this reason. Likewise, pp11 said they chose to focus on the sound of the crosswalk signal instead of carrying on squinting at it. Pp07 also discussed the effects of bright light on their energy levels. They said that bright lights automatically grab their attention, while they should actually focus on the road. Lastly, something that came up multiple times is the decision to take a (regional) taxi instead of public transport on either their way to or from a goal location as a way to conserve their energy.

#### 3.7.2. Emotions and Opinions

Participants referred to various emotions and opinions that come up for them during wayfinding. With regard to their own emotions, anxiety-related codes were the most prominent in the data, including anxious, tense, insecure, and careful. Especially, pp09 talked about their anxiety about navigating busy environments with a lot of traffic. For pp01, the consequence of their anxiety is that they avoid walking certain routes that they used to walk in the past. More positive emotions that came up were pride, curiosity, pushing one’s own boundaries, and optimism. Other emotions and opinions that were mentioned are a sense of responsibility, feeling ‘different’, feeling dependent, perseverance, acceptance, patience, and loneliness.

Participants also commented on the influence of the reactions of their loved ones on their navigational behaviour. For example, it can be the case that someone is trying to protect their visually impaired loved one’s energy levels by discouraging particular activities, such as going to the supermarket by themselves.

#### 3.7.3. Needs

Participants also mentioned navigation-related needs during the interviews. The most notable needs were a need for independence (most frequent), the need for shorelines or directional tactile paving, and the need for security. The need for independence is expressed by VIPs denying any help that is offered and continuing their journey on their own, or choosing to use smartphone apps to travel somewhere autonomously. Whereas some individuals prioritise independence, others can find this a waste of time or energy and instead prioritise efficiency or convenience by asking for help. Other participants value independence conditionally (e.g., on the condition that they are not walking with their dog). The need for shorelines is mentioned only by the cane-using participants and is evident in VIPs’ explanations that certain parts of their routes would have been easier to navigate autonomously if there had been shorelines (e.g., open spaces or busy environments), and in the fact that they avoid environments that lack proper shorelines. The need for security is implied by the number of checks that VIPs implement along their route to verify their judgments (e.g., during public transport), or by the variability between VIPs regarding the need to feel well-prepared before embarking on their journey.

#### 3.7.4. Attitude Towards Mishaps

Our participants exhibit different attitudes towards things going wrong during wayfinding. The attitudes that we found are accepting (i.e., actively accepting that making errors just happens every now and then, or putting it into perspective; pp01, pp07), perseverant (i.e., wanting to try and keep trying when mishaps occur; pp07, pp09), indifferent (i.e., not feeling one way or the other about a mishap; pp02, pp07, pp08), fearful (i.e., feeling tense or anxious when the risk of making errors occurs; pp07), avoidant (i.e., making conscious decisions as to avoid mishaps; pp01), and audacious (i.e., being animated about making conscious decisions that increase the risk of mishaps; pp08). For several participants, it has been a deliberate learning process to accept that mishaps will occur during navigation, build the self-confidence to deal with them, and accept help from others (pp01, pp05, pp07, pp09):

“*Also accept that things can sometimes go wrong, because I think that is a very important one: accept that things can also go wrong and especially don’t blame yourself. Yes is a, yes, I think, I think that every blind person in the beginning- that’s what I think, or it must be just me, I always got angry a lot in the beginning, like “watch where you’re going, man!”, while you actually couldn’t.*”*—pp01*

### 3.8. Navigational Intention

Navigational intention is the second of the two main themes that influence cue use and strategy choice. Navigational intention describes how the navigation-related intention that a VIP has at a certain point along a route can influence which cues or strategies they prefer. The Navigational intention theme is subdivided into Orientation, Mobility, and Safety, which means that certain sensory cues and strategies are chosen to fulfil either an orientation-, mobility-, or safety-related goal.

**Figure 5:**
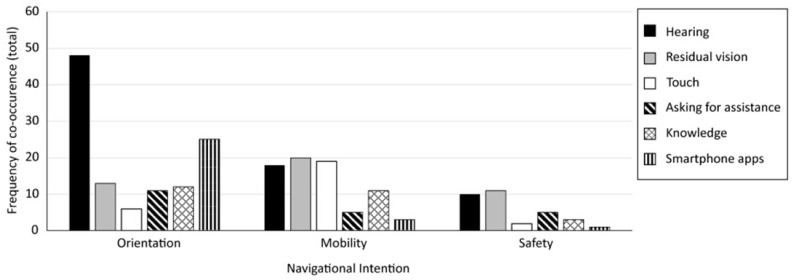
Strategic use of available navigational information. This figure shows how often the codes for Asking for help, Knowledge, and Smartphone applications, and the most frequently mentioned sensory modalities (i.e., which source of information is employed) occur in combination with the orientation, mobility, and safety codes (i.e., which navigational goal is meant to be reached at a certain time and location).

Figure 5 shows the frequency of co-occurrence in the data between navigational intention (i.e., orientation, mobility, or safety) and the source of information chosen (i.e., either one of the three most frequently mentioned sensory modalities, and the codes for Asking for help, Knowledge, and Smartphone applications). These findings suggest that the participants in this study tend to employ different sensory modalities for different purposes while navigating. Specifically, sound cues might be most helpful when trying to orient oneself compared to visual or touch cues. Touch cues might mainly be beneficial for mobility purposes, though residual vision, hearing, and touch might be equally favoured for mobility purposes. For safety purposes, only residual vision and hearing might be useful. Furthermore, the data suggest that smartphone applications are mostly used for orientation rather than mobility or safety purposes, especially in unfamiliar environments. Lastly, asking for help is a strategy that is mentioned predominantly in the context of orientation, and the strategy of using pre-existing spatial knowledge is mentioned in the context of orientation as well as mobility.

#### 3.8.1. Orientation

Participants gather or use spatial information with several different intentions in mind, one of which is orientation. In the data, all instances were coded as orientation in which a person spoke about knowing where they are within an environment, about making a wayfinding choice with the purpose of progressing along their chosen route, or about knowing where to go next during route-following.

*Landmark*. The Landmark code was applied to all data in which a participant discussed using a certain cue as a means of recognising where they are along a route or within an environment. Most cues that participants discussed were found in their immediate environment (i.e., local landmarks). However, participants also reported using the distance of a cue as an orientational strategy. Examples of this are distal landmarks, such as using the sound of a railway in the distance to orient oneself during a walk. Furthermore, there were a few instances in which a landmark was used for route progression. For instance, some cues were used as ‘directional’ or ‘associative’ cues [63,64], i.e., recognising a landmark is used to judge when to change directions (specifically, knowing when to take a turn to the left or right). In other instances, cues were used as ‘maintenance’ cues [65] or ‘reassurance points’ [44], i.e., the recognition of a landmark results in the judgment that the navigator is still following the desired route. A final instance is that cues were used as beacons [63], i.e., the navigator knows that moving directly towards a certain cue will result in progression along their route.

*Fine-grained navigational goal.* The Fine-grained navigational goal code is formulated to refer to instances in which a person had to reach a specific goal during route-following and that goal is not necessarily related to following the route itself. Usually, these goals are not larger than the person themselves. Examples of fine-grained navigational goals that are sought out are small gates or doorways that must be travelled through or buttons that need to be pressed (e.g., buttons to open the train door, or doorbells) in order to be able to progress towards a desired end location.

#### 3.8.2. Mobility

Besides orientation, participants also discussed navigational strategies concerning mobility. In the data, the Mobility code was applied to a text fragment when a participant spoke about the aspects of route-following that concerned the relationship between their body and their direct environment.

Obstacles. One aspect of mobility is the ability to avoid obstacles in their way in order to be able to safely resume their route. Some obstacles simultaneously serve the purpose of a landmark, as the object is supposed to be avoided, but also give the navigator information about which point they are at along their route.

Keeping to a path. The sub-theme Keeping to a path includes the ability to walk in a straight line, as well as the ability to follow one specific path, including the bends. Examples of this are participants describing the use of shorelines, such as gutters.

Steps up and down. The Steps up or down sub-theme is about all instances in which participants mention perceiving or reacting to steep elevation differences in the walking surface. Examples include managing curbs and stairs.

Width of a path or road. Observing the width of a path or a road was primarily mentioned by participants who have or have had a guide dog. Namely, they mention needing more space when walking with a dog, which results in them paying attention to this mobility aspect.

Positioning. The sub-theme Positioning refers to the instances where our participants specifically mention the side of a street they are following, or their relative place on a road or path:

“*If I walk on the wrong side of the road, you often have these parking bays, or then you have to- it’s a bit of a narrow sidewalk, or, or you know, that kind of thing. So if I know my way around and I know the best side of the street to walk, then I always take the easiest side, of course.*”*—pp11*

#### 3.8.3. Safety

The third sub-theme of Navigational intention is Safety. The Safety code was applied to text fragments in which participants talked about things like watching out, being careful, feeling safe, being safe, or avoiding accidents or harm to oneself or others.

*Intersections*. Our participants mainly talked about intersections in the context of safely crossing roads and avoiding collisions with other road users, e.g., by explicitly using one’s hearing in addition to residual vision in order to safely cross a road:

“*I am careful, though. Because I also know from experience, crossing a pedestrian crossing is not a guarantee that you will survive. So I do also try to look left and right, and to listen: is something there? Because also, yeah, what I, yeah, there it is again, cyclists. I hardly ever hear those.*”*—pp09*

*Tripping hazards*. Another important safety aspect is tripping hazards. This sub-theme partially overlaps with Steps up or down, though steps up or down are not always referred to as tripping hazards. Other possible tripping hazards that were mentioned are loose tiles, tree roots, or small fences. One participant (pp11) even made safety an explicit point to talk about, introducing the topic of tripping hazards at the start of the interview. When all protocol questions were asked and the interview was concluded, the final protocol question was asked (i.e., asking the participant whether they would like to add something). They then reminded the interviewer: “shall I describe some situations in which I almost tripped?” An example of such a situation is when they wanted to cross a road that consisted of a two-lane road as well as two cycling paths. They were paying attention to the passing traffic and missed an unexpected curb separating the cycling path from the road, causing them to trip and fall onto the road.

## 4. Discussion

This study explored wayfinding experiences of people with visual impairments (VIPs) via qualitative interviews in which participants were asked to describe a familiar route in their daily lives. The main findings show that VIPs prefer gathering information during wayfinding through using their senses (mainly hearing), employing existing spatial knowledge (mainly knowing the layout of an environment), asking or receiving assistance from other people (mainly walking along during a segment of a route), and use of technologies (besides the white cane mainly via auditory-verbal instructions from smartphone applications). The way VIPs report gathering sensory information seems to be influenced by the intended navigational goal that needs to be achieved at a certain time and place (i.e., an orientation-, mobility-, or safety-related, or fine-grained goal), environmental factors (such as weather conditions), and affective factors (such as personal priorities pertaining to independence and energy management).

Besides the reported information gathering strategies, we identified specific cues that our participants seem to find most salient during navigation. These include pedestrian crossings and traffic, acoustics (including echoes), stairs, the behaviour of a VIP’s guide dog (used as an indicator to orient oneself), various types of shops and restaurants, and directional tactile paving or shorelines (i.e., linear boundaries such as sidewalks and walls, but also grassy edges, gutters, and fences). These findings are generally consistent with previous studies on salient navigational cues for VIPs, especially traffic and shops [22,38,39,40,41,43,44,46,66]. Notable exceptions include a guide dog’s behaviour and acoustics. Our analysis revealed that a guide dog’s behaviour and acoustics can be useful non-technological wayfinding cues for VIPs, while this has not been highlighted yet in existing literature. The emphasis of our participants on guide dog behaviour may reflect the relatively high prevalence of guide dog users in the Netherlands compared to the countries represented in the literature [67]. Regarding acoustics, the discrepancy might be a result of our method of analysis. Acoustics are not often treated as a type of cue, but as a derivative of particular cues. Examples of such cues are the ‘feeling’ of high buildings or open spaces such as parking lots, both of which have occurred in earlier studies. Conversely, while we did identify trees as a useful navigational cue, it was one of the least frequently mentioned cues, whereas previous studies included them among the most useful cues. This suggests that, although our participants might implicitly or explicitly notice trees along their routes, other cues are more salient to them. This might be due to the potentially greener urban environments of our participants, resulting in trees not standing out within the environment as a salient feature. For guide dog users in particular, trees might be experienced as obstacles that their dogs avoid rather than as useful wayfinding cues. Cane users might favour shorelines because they tend to be more consistent and continuous, whereas trees could be less linear or predictable.

To perceive and use these cues during route-following, our findings suggest that VIPs focus more on their sense of hearing compared to their sense of touch via cane use, feet, or hands. When taking a closer look at the context, when hearing and touch are preferred, we see that this depends on the navigational goal at a given time and place. That is, we found that hearing is mostly mentioned in the context of orientation purposes (i.e., determining where I am and where I need to go, for example, by identifying landmarks) as well as safety purposes (e.g., using sounds as a go/no-go signal when crossing a street). This contrasts with previous findings by Wang and colleagues [46] that show touch instead of hearing is experienced as most useful for navigation. Wang and colleagues were specifically interested in landmark use. The landmarks reported in their study include mostly local landmarks, such as walking surface textures or the lowering of a sidewalk, rather than more distal landmarks. This might be an explanation for why they found a dominance of the haptic modality, as touch only provides information about one’s immediate surroundings, as it requires direct contact between the navigator and the environment. Indeed, when taking the type of landmark into account, we find similarly that the local cues we identified are often primarily perceived by touch (i.e., similarly, a change in walking surface texture or the lowering of a sidewalk, but also cues such as feeling a specific street planter). Still, in general, we found hearing to be the dominant sense for navigation. An explanation for this could be that hearing, besides vision, might be the only sensory modality that provides real-time directional information about both the immediate and the broader environment. Auditory cues could therefore be uniquely suitable to identify distal landmarks to determine one’s orientation in relation to their understanding of the layout of a broader environment, rather than immediate navigational decisions only.

Though hearing was the most frequently reported sensory modality, it varied between participants in which sensory modality they preferred and which navigational goal they primarily focused on. It seems that participants in our sample with the most residual vision left also tend to rely on their vision more compared to the other VIPs while navigating (pp07, pp09, pp11). They also seem to be mostly preoccupied with goals related to mobility (e.g., not bumping against a curb when cycling) and safety (e.g., managing tripping hazards), rather than orientation (e.g., determining the right direction, or figuring out when to take a turn). Three other participants relied mostly on touch (pp01, pp04, pp08). Notably, these are the participants who have never navigated with the help of a guide dog and only use a cane for orientation and mobility, seemingly preferring shorelines and directional tactile paving. Yet other participants focus a lot on their hearing (pp02, pp05, pp06), with acoustics and echolocation standing out to orient themselves. Two participants (pp03, pp10) did not seem to have an explicit preference in terms of sensory modality, though hearing was their most frequently mentioned sense. Our analysis suggests that none of the participants relied greatly on olfaction or vestibular proprioception for wayfinding. However, we did find that some participants did sporadically report olfactory cues as helpful (e.g., using the smell of a certain shop as a landmark), while others did not mention them at all.

We found that which sensory modality or other resource is chosen not only depends on current navigational goals, but also on environmental factors. Specifically, we generated the sub-themes Environment, Familiarity, Weather conditions, and Crowdedness to capture environmental factors influencing resource choice. Comparatively, Williams et al. [24] distinguished the sub-themes Terrain, Familiarity, Weather, Crowd density, respectively, within their Scenario theme. In contrast, though, Williams and colleagues also included Transportation availability and GPS availability as sub-themes. The former, Transportation availability, is about the accessibility of public transportation. It makes sense that this was more of an issue for residents living in the United States of America as opposed to Western Europe, due to fewer accessible public transit options [68,69]. The latter, GPS availability, is about the availability of GPS in indoor or “off-grid” environments compared to regular outdoor environments. While the indoor/outdoor distinction was of importance to our sample too, this was not classified in our study as an issue due to GPS availability reasons, per se. Instead, it seemed more appropriate in our thematic framework to classify the difference in difficulty between navigating (unfamiliar) indoor environments and outdoor environments as the higher-level theme, with the limited existence and usefulness of navigation apps (and thus GPS-related technologies) in indoor environments as just one of the influencing factors.

To the question of why VIPs prefer one type of resource over the other and why certain environmental factors matter to them, we conclude that a lot of it comes down to energy management. Deliberate wayfinding choices regarding route and means of travel are necessary in order to preserve sufficient energy, depending on what a person prioritizes (whether it be the activity at the destination itself or a feeling of independence). Specifically, crowded, indoor, unfamiliar environments, and brightly lit environments all negatively impact VIPs’ energy levels as they require more attention from the navigator and hence increase the information processing load of the wayfinding task at hand. First, unfamiliar environments have higher cognitive costs due to uncertainty regarding orientation, mobility, and safety compared to familiar environments. In terms of resource choice, unfamiliar environments often call for smartphone use to orient oneself, as well as more assistance from other people to reach desired destinations. Smartphone applications and Other people are partially used for the same purposes: for preparation, for confirmation in uncertain circumstances (e.g., when lost, or as an extra check during public transport), or as an extra help to know where to go (i.e., in addition to sensory modalities). From these findings, we conclude that Other people and Smartphone applications are turned to when Sensory modalities and Knowledge provide insufficient information to confidently fulfil a navigational goal. Second, crowded areas are also more energy-consuming than quiet environments, due to more focus being necessary to pick out one’s auditory points of reference or traffic flow for safety purposes. On the other hand, the sound of, e.g., crowds, traffic, or individual people can be experienced as useful cues for orientation. However, it varies per person whether they prefer to switch to primarily using their sense of touch in crowded areas, versus still focusing on particularly salient auditory cues such as loud recognisable voices.

The insights generated in the present study could be of importance to occupational therapists, app developers, policymakers, urban planners, or accessibility consultants. For O&M trainers, mapping the personal preferences of their clients might inform which routes or cues might be favoured during training [70]. Consequently, O&M trainers could design their O&M training sessions taking into account the individual’s preferences, as well as knowing which characteristics to monitor extra closely during training. For app developers, the categorisation of VIPs’ wayfinding preferences can be used to assess which types of cues would be useful or desirable to incorporate in certain technologies, to decide which features are best suited to make customizable, or to improve the perceived trustworthiness of the app [21,71,72]. Finally, assessing VIPs’ navigational preferences and challenges can be informative for policy-making regarding the design of accessible public spaces, both outdoor and indoor [20,22,30,73]. It should be noted, however, that subjective reports of wayfinding strategies may not always align with individuals’ objectively observable navigation behaviours or with the actual impact of these preferences on wayfinding competence. Future research that systematically links subjective accounts to behavioural or performance-based measures would further clarify these relationships and strengthen the practical implications for O&M instruction, technology design, and accessibility planning.

The current findings shed light on the wayfinding preferences of VIPs residing in the Netherlands. Therefore, the findings are not completely generalisable to VIPs in countries with different infrastructural, cultural, or environmental contexts. Nonetheless, we think that the themes and many of the subthemes identified in this study apply to other countries and societies as well. The current findings might partially extend to other countries that share some of the Dutch characteristics. Such characteristics would include white canes and guide dogs as primary navigation aids for VIPs, as well as relatively densely built, high-entropy urban areas with tactile paving and relatively reliable, well-connected, and accessible public transit networks (including free smartphone applications and websites with real-time travel information).

Though this study purposefully included VIPs with varying types of impairments, levels of residual vision, ages of onset, and types of navigation aids, the findings do not allow us to draw definitive conclusions about differences in cue and strategy preferences between these groups. For instance, variations between individuals regarding wayfinding preferences might be found between cane users versus VIPs with guide dogs. Moreover, we excluded VIPs with comorbid conditions, such as hearing loss or intellectual disabilities. These subgroups likely have unique wayfinding experiences compared to subgroups with visual impairments alone, so it might be the case that additional or different preferences may emerge among VIPs with impairments not represented in our sample. In addition, the study’s participants were mainly recruited through a pool of VIPs who had signed up to volunteer as participants in research projects. As a result, our sample might overrepresent individuals who lead relatively active lifestyles and travel more frequently or over longer distances than a typical VIP in the Netherlands. However, it is known from previous studies conducted in several countries that 20 to 30 percent of the VIP population rarely leave their house to travel somewhere [45]. So, a possible downside of our recruitment method is that we might have missed the categorisation of needs, preferences, and motivations specific to VIPs who choose to make fewer journeys or prefer to stay in more familiar environments. These limitations will be addressed in an upcoming quantitative study including a large, diverse sample of VIPs. While the current study provides an overview of the types of wayfinding preferences that can be found within this population, a future study would be aimed at mapping individual differences by classifying these wayfinding preferences in the context of a range of personal characteristics, impairment-related characteristics, and type of mobility aid used.

Finally, some participants participated in O&M training during their lifetime, whereas others did not. This variation in experience with O&M training might have caused variations in the richness of the data. Namely, VIPs who do have experience with O&M training may have a larger number of navigational strategies in their toolbox, and might possess greater awareness of their preferences and strategies compared to untrained individuals, resulting in an increased ability to articulate which cues they focus on during route following. This could have led to less detailed route descriptions of participants who did not follow O&M training compared to participants who did. This hypothesis is partially supported by findings in a follow-up study, in which O&M instructors were interviewed and described how they actively coach their visually impaired clients to utilise non-visual sensory information for orientation, mobility, and safety purposes [74]. In this follow-up paper, we conclude that training generally seems to follow a four-step structure (i.e., teaching a VIP strategies to notice, interpret, act upon, and anticipate relevant sensory cues). We also address individual differences between VIPs that O&M instructors take into account during training.

## 5. Conclusions

We found that hearing is the most important sensory modality to VIPs for orientation purposes, although it varies per person how often other resources (i.e., other sensory modalities, existing knowledge of an environment, help from others, or navigational aids) are relied upon. These strategic choices seem to depend on environmental factors (such as weather conditions, crowdedness, and familiarity of the environment), which are possibly mediated by individual differences in priorities or needs pertaining to energy management. Our thematic analysis not only provided insight into the most important factors in navigation for people with VIPs, but also provided a structure to think about navigation with no or limited vision. This structure is useful for multiple fields related to navigation, like architects and designers working on improving navigation in situations with limited visual information, app developers, and urban planners.

## Figures and Tables

**Table 1:** Overview of the demographics of our sample. All information is self-reported.

Participant	Aged Between	Main Diagnose(s)	Onset of the Impairment	Current Residual Vision (Left; Right) and Visual Field Description	Type of Aid(s)	Public Transport Activity	Additional Symptoms
01	60–70	Glaucoma; Retinal detachment	Late onset (diagnosed at age 57)	None; None	White cane (previous: symbol cane), smartphone, camera glasses, wireless keyboard	Makes use of taxis. Avoids buses.	Sometimes fully black vision, sometimes fully white vision
02	50–60	Glaucoma; Corneal abnormality	Congenital	None; 1/300 ellipse shaped visual field, very blurry	White cane, smartphone (previous: guide dog)	Travels by train and bus. Avoids taxis.	Light sensitivity
03	40–50	Bilateral anophthalmia	Congenital	None; None	White cane (previous: guide dog)	Mainly travels by train, sometimes by bus.	None
04	40–50	Diabetic retinopathy	Late onset (diagnosed at age 37)	None only light-dark; 3–5%, spots in upper visual field	White cane, guide dog, smartphone, contrast glasses	Travels by bus and train. Only uses taxis in unfamiliar environments.	Less sensitivity in foot soles
05	40–50	Retinal detachment	Early onset (diagnosed at age 15)	1%, only light-dark, very blurry; None	White cane, guide dog, smartphone	Mainly travels by bus, and sometimes by train.	Light sensitivity
06	70–80	Glaucoma; Pigmentary retinitis	Congenital with progressive decline	None; None	White cane, guide dog, smartphone, reading glasses	Makes use of trams, trains, buses and taxis.	None
07	50–60	Nystagmus	Congenital	12–15%; 12–15%	Smartphone, loupe glasses	Drives a light quadricycle and rides a bicycle (weekly), and sometimes travels by train (a few times a year).	Light sensitivity
08	30–40	Cataract; Optic nerve damage (right eye)	Congenital	0,2%, tunnel vision; None, only light-dark	White cane (ball tip)	Travels by bus and train.	None
09	60–70	Vasculitis (right eye); Retinal detachment (left eye); Foveal damage (left eye)	Late onset (diagnosed at age 55)	10-12%, blurry, tunnel vision; None	White cane, smartphone	Travels by bus or train (almost daily).	Light sensitivity
10	50–60	Retinitis pigmentosa	Congenital with progressive decline	0,3%, only light-dark; 0,3%, only light-dark	White cane, guide dog, smartphone, cap, sunglasses	Travels by bus and train (a few times per month).	Light sensitivity
11	50–60	Cone dystrophy	Late onset (diagnosed at age 50)	0% central, 10% periphery; 0% central, 10% periphery	Smartphone, cap, sunglasses	Travels by train (once every two months), and sometimes by tram or bus.	Light sensitivity

## Data Availability

The data presented in this study are available on request from the corresponding author due to privacy.

## Abbreviations

The following abbreviations are used in this manuscript:

VIP	Visually impaired person
O&M	Orientation and mobility

## Appendix A. Interview Protocol (Translated)

Preliminary Information

[Introduction mentioning that we have already spoken on the telephone and asking about the information letter] Have you had a chance to read through it?

- [If no (not read):] Read the letter aloud.
- [If yes:] That’s great, thank you. I would now also like to provide some information verbally, and then see if you have any questions, is that all right?

Over the next hour, I will ask you questions about how you navigate. This can be both indoors and outdoors. Through this conversation, we want to find out how you prefer to use your senses when trying to get from A to B, particularly your sight [if applicable], hearing, and touch. I personally find it very interesting to investigate how our senses work (together) and how this can differ from person to person. We will use this information in the research to see if we can distinguish the individual ways and preferences during navigation. We then want to use that knowledge during, for example, mobility training. Is it clear what the research involves, or do you have any questions about anything?

It was also mentioned in the information letter that it is necessary to record the conversation so that we can listen back to what people have said exactly. Is it all right with you if the conversation is recorded?

- [If yes:] I have a consent form here which states what you are giving consent for if you participate in this study, and you can give your consent by putting your name on the form just above where you feel the set square on the form. Would you mind reviewing the form? If there are any questions or comments, I would be happy to hear them.
  ○ [Read aloud if necessary.]
- [If no:] Would it be all right if I wrote down your answers instead of recording?
  ○ [If yes: Write down the answers as accurately as possible and continue with the text below].
  ○ [If no: reproduce the data yourself as accurately as possible afterwards.]

Thank you very much. Then we can get started in a moment. But first, I want to emphasise that there are no right or wrong answers in this interview. We are here because you have expertise and important knowledge that can help science and other people with visual impairments. We are purely interested in your experiences with navigation, and you can certainly mention things that seem very obvious to you. Sometimes I will write things down; that is for myself for when I will analyse all the interviews, for example if I get an idea for the analysis or hear something that reminds me of something else that has been said before.

Do you have any questions or comments before we begin the interview?

- [If yes: answer questions.]
- [If no:] If any questions come up, you can ask them at any time. I will now turn on the audio recording and we can get started! Are you ready?

Introductory Questions

1. Could you start by telling me something about yourself—who are you, what do you do in your daily life?
2. Have you ever participated in an interview before?
  - [If yes]: Do you remember what kind of questions you were asked then?
  - [If no]: Right, a new experience! Exciting?
3. So, today’s interview is about navigation. Would you like to tell me something about what your week looks like, specifically the days when you go outside to go somewhere such as work, hobbies, friends, shops, events?

Transitional Questions

1. To which of these occasions do you travel yourself on your own using public transport?
  - Choose a location; this becomes location X in the follow-up questions.
  - If no journeys are made using public transport, replace ‘public transport’ with the alternative means of independent travel (walking, bicycle, scooter). Skip Substantive Questions 2 and 3.
2. When was the last time you travelled to a new location on your own using public transport?
  [Possible follow-up questions:]
  - Where were you going then?
  - How long ago was that?
  - How often do you travel to new locations?
    - If a complete answer is given immediately: Right, completely clear, thank you.
    - If there is no recollection, ask follow-up questions:
      1. Is there a reason why you don’t often go to new locations on your own using public transport?
      2. Would you like to travel to new locations on your own?
        - Why/why not?
    - What would make it easier for you to travel to new locations?

Substantive Questions

1. Now I would like to ask more questions about more familiar journeys for you. You said earlier that you travel to X weekly using public transport. I would like to go through your journey to X with you in the upcoming questions, is that all right?
  - [If yes: continue.]
  - [If no: choose another location together.]
2. Imagine you are planning to go to X. What does the preparation for that look like from the moment you decide to go to X until the moment you want to step out of the door?
  [Possible follow-up questions:]
  - What kind of things do you take with you?
  - What kind of information do you look up about the route(s), journey, or location(s)?
  - How does the preparation for unfamiliar routes or locations differ compared to familiar routes or locations?
  - How do you look up that information?
  - How do you find your way to the front door?
3. And then the journey to the station or bus stop. You step outside. Can you take me through what that is like for you, what are things you pay attention to when navigating there?
  [Possible follow-up questions:]
  - What are things you see (if applicable)?
  - What are things you hear?
  - What are things you feel?
  - Is there any other information that you haven’t mentioned yet that you use to find your way or know where you are?
  - How do you find your way to the correct train or bus?
4. Then the train/bus arrives. Can you tell me…
  - …how you find your way to the doors?
  - …how you handle your ticket?
  - …how you find an empty spot?
  - …what you pay attention to during the bus journey?
    - Sight
    - Hearing
    - Touch
  - …how you know you are at the correct stop to get off?
  - …what you do when you have reached the correct station or stop and want to get off?
5. When you have gotten off, you are in a new environment and want to make your way to the new location [if public transport]//You arrive in the area of X and want to make your way to the destination [if walking/cycling/scooter].
  Would you mentally recall as vividly as possible for me how you experienced that route from the stop to the location, and can you tell me what you paid attention to during your journey from the stop to the location to find and maintain the right way?
  [Possible follow-up questions:]
  - What are things you see on the way to X (if applicable)?
  - What are things you hear?
  - What are things you feel?
  - How do you find your way to the entrance?
6. And then the last part of the journey: you have arrived at location X, and then still need to get to the correct room within X. How do you approach that?
7. Suppose that at some point you have taken a wrong turn on the way to X. How would you notice that?
  [Possible follow-up questions:]
  - How would you try to get back on the right route?
8. Would you say that you have a preferred sense?
  - If yes: which one and why?
  - If no: why not?

Closing Questions

1. An hour has now passed, and that means we have come to the end of the interview. Are there any matters that you think we have missed, or are there other things you would also like to share?
  - [If yes: let participant make additions].
  - [If no]: Shall we end the interview then? I will now turn off the audio recording and would like to thank you very much for your time and participation!
  Conclusion
  [Give a summary of the main points talked about.]
  We will create an anonymised text file from the audio recording and then analyse it together with the text files from other interviews to see if we can discover certain patterns.
  When we have completed all the interviews, we want to share the results in a newsletter by email. Would you also like to receive that newsletter? This would be a one-off newsletter. It is not yet known exactly when it will be sent, but it will certainly take several months, as we still need to process the results.
  - Are there any other things about the research that are not about the content that you still want to mention?
  - How do you feel/How did you experience being interviewed?
  [After completion, researcher updates their reflexivity journal and field notes.]

## Appendix B. Case Summaries

In this appendix, short summaries of each of the participants are presented to illustrate how the (sub-)themes generated in this study can interact within a person. To keep the data anonymous, ages are given in ranges, and gender indications are left out. Concepts relating to themes and sub-themes are underscored for emphasis.

### Appendix B.1. Case 01

Participant 01 (aged between 60–70 years old) became totally blind relatively late (diagnosed with glaucoma at age 57 and retinal detachment at 59 years old). The participant started out using a symbol cane but transitioned to a white cane when they became totally blind. In their daily life, this participant does all kinds of visual impairment-related volunteering (e.g., advocacy, testing out products, and being an expert by experience). The participant preferred to do the interview at an institution for people with visual impairments, as the participant could combine this with another appointment there. The participant came across as a person with an optimistic outlook on life. It was important to them to refer to their impairment as a visual challenge instead of a visual impairment, because, in their own words, the participant ‘looks at chances and opportunities’. During the interview, they stressed that they prefer a slow walking tempo because they like to consciously focus on their different senses and wish to gather the same amount of information about their environment as sighted individuals. This was also apparent from their very grateful reaction to the interviewer asking at the beginning of the interview whether the participant would want to know who was sitting in front of them by describing what the interviewer looked like.

Themes that seemed relatively important to this participant were a strong need for independence, a need for freedom, and the need to fully experience the environment. It was very important to this participant to use all of the senses to gather information (with the mention of the sense of touch and the sense of smell relatively often), even when it is not necessarily important for orientation, mobility, or safety. Examples of this were them describing the material of the table in front of them, using echolocation to gauge the spaciousness of the room we were sitting in, and describing how they identify flowers when taking a walk. Furthermore, they talked a lot about which needs they had during wayfinding and any emotions that might come up for them, such as the happiness and pride the participant felt when he noticed a slope that they could use to take out the trash by themselves, or the enjoyment of purposefully taking new routes or shortcuts to get themselves more acquainted in a given environment. Lastly, the white cane was also very relevant for this person, whom they explicitly like to use to identify objects. This participant also tends to use characteristics of the walking surface texture to orient themselves.

### Appendix B.2. Case 02

Participant 02 (aged between 50–60 years old) is severely blind due to a congenital corneal abnormality as well as later acquired glaucoma. This participant has experience with having a guide dog and has had a white cane for as long as they can remember. In their daily life, the participant is a homemaker and does a lot of volunteering with and for visually impaired people (e.g., at the Eye Associations, the Eye Café, and a Christian library for blind individuals). They can also read Braille. The interview was held at the participant’s home, which was tidy and spacious. The participant tended to go through their descriptions of their routes relatively quickly, resulting in the need for frequent interruptions during the interview to ask further questions.

For participant 02, their guide dog was essential in their navigation experience compared to other participants. Something that was of interest is that they often mentioned using their guide dog to find fine-grained navigational goals, which are generally experienced as difficult points during wayfinding. This participant also emphasized several benefits of having a guide dog. From their route descriptions, it also became clear that the participants use their knowledge of an environment, sense of hearing, and smartphone apps when navigating. The participant predominantly uses their smartphone in moments of doubt, to double-check where they are or needs to go. This participant virtually never mentioned obstacle avoidance, so this was apparently not of their concern. This makes sense considering a guide dog is often dubbed “obstacle avoider”. This person also focused on asking acquaintances and passersby for help with regard to navigation.

### Appendix B.3. Case 03

Participant 03 (aged between 40–50 years old) was born without eyes. This was noticeable in their body language (e.g., the face but not the body facing the interviewer when conversing). The participant has a now-retired guide dog (though the participant is currently in the process of getting a new one), and they have been using a white cane their entire adulthood. The participant can also read Braille. The participant was of slight build and came across as a relatively reserved person. In their daily life, they have many sportive and creative hobbies. The participant preferred doing the interview at home. They kept their answers short and to the point, resulting in multiple silences and the need for frequent probes during the interview.

A preference to travel together or traveling with specialized (individual) transport came up a lot. The participant told the interviewer that it can be convenient to have other people take you places instead of being self-dependent all the time, as it is important to them that the energy they save by doing so can be used for the actual hobby or event itself. In terms of route-following, the participant focused comparatively often on directional cues and acoustics, and comparatively little on shorelines or directional tactile paving. However, the participant does talk about sensing differences in walking surface texture. Thirdly, they talked relatively often about the disadvantages of guide dogs. Lastly, this participant expressed difficulty with using navigational apps, even though they would really love to be able to use them.

### Appendix B.4. Case 04

Participant 04 (aged between 40–50 years old) has had a mild visual impairment (due to diabetic retinopathy) since age 29, but became severely blind three years prior to the interview due to a diabetes-induced coma. In their daily life, this participant does a lot of impairment-related volunteering (e.g., as a host, in the municipality, and participating in research). This participant also spends their time growing pumpkins with their spouse, and told the interviewer that their spouse always says that they are better at telling when a pumpkin is ripe compared to their spouse because of using the sense of touch so well. Furthermore, this participant has experience with traveling with a guide dog in the past (for four years), but currently only uses a white cane (since age 31). They also wear glasses with yellow-amber lenses. Participant 04 came across as a cheerful and energetic person. The interview was held at an institute for people with visual impairments, where the participant is a volunteer. Afterwards, the participant proposed to walk the interviewer to the train station and show the cues they use while navigating (i.e., the route also described during the interview).

This participant is a self-proclaimed proficient cane user. This also became apparent in how often shorelines or directional tactile paving were mentioned when describing their routes, which the participant observes with their cane. Their focus was largely on orientation (i.e., determining their location or next destination). Besides using shorelines to follow their route, the participant also talked a lot about landmarks that they use to orient themselves, and relatively often about using smell for orientation. Their focus was not so much on mobility issues.

### Appendix B.5. Case 05

Participant 05 (aged between 40–50 years old) suffers from hereditary retinal detachment. The participant has been very severely blind for fifteen years in the left eye (the right eye has been replaced by a glass one at age 4), and has had a guide dog since age 22. This participant also has a white cane, though they do not prefer using it (especially when walking the dog). This participant came across as a bubbly and talkative person. In their daily life, this participant has many hobbies (sports, reading, and music), and does a lot of volunteering (both impairment-related and other types of jobs). However, the participant has also spent six years at home raising their children. During this time, the participant has not often left the house, which has been difficult for them. The interview took place at the Utrecht Science Park.

Participant 05 was a special case in the sense that it was the only person that talked at length about a plethora of sensory cues they come across when walking routes. This participant focuses mostly on their hearing (in particular, using acoustics and echolocation skills), though olfactory and vestibular cues also came up relatively often. Furthermore, this participant talks often about combining cues and using knowledge of an environment, including its landmarks, intersections, and roundabouts. There was also a large focus on the guide dog, even during the interview itself they commented multiple times on the dog’s behaviour. The participant sometimes prefers traveling with other people to get somewhere, but these are usually cases in which their dog cannot come with them.

### Appendix B.6. Case 06

Participant 06 (aged between 70–80 years old) is the oldest participant, though they looked young for their age. This participant has been diagnosed with retinitis pigmentosa and glaucoma, has been visually impaired since birth, and has been totally blind since age 58. Since that age, this participant has been travelling with a white cane and a guide dog (currently has two guide dogs, one of which is retired). In their daily life, the participant has sports-related hobbies and has multiple impairment-related volunteering jobs, one of which focuses specifically on public transport in the area. This participant came across as a ‘no-nonsense’ type of person.

This person has an interest in navigation, and especially in different types of technologies like GPS-systems and smartphone applications: mostly using the auditory-verbal instructions for knowing when to take a turn left or right. This participant also talked at length about the various navigation systems they have used in the past, while taking them out of their drawers to show them to the interviewer. This person’s focus was on all kinds of needs they have with respect to wayfinding and mobility. Mainly, their preference for smartphone apps is related to their need to be as independent as possible. Notably, this participant has an explicit preference for an indoor navigation app rather than a guiding line. Furthermore, their attitude was very different for familiar routes versus unknown areas, describing familiar routes as ‘a piece of cake’, though in unknown areas, they like to travel together with an acquaintance or ask assisting personnel for help. This participant also talked relatively often about their sense of hearing when describing their routes. This participant even uses their hearing in crowded areas, in contrast to other VIPs, picking out sounds that are salient to them and using those as points of reference.

### Appendix B.7. Case 07

Participant 07 (aged between 50–60 years old) has a congenital moderate visual impairment (nystagmus due to Oculocutaneous Albinism) and does not use a cane or a guide dog. In their daily life, this participant works in home care, and in their free time, they go to the gym and occupy themselves with various social activities. This participant was the only one who reported driving a vehicle (i.e., a light quadricycle) and described themselves as being totally independent. The interview took place in the participant’s home, in which they moved around in a noticeably more agile way than the interviewees with less residual vision. The participant came across as an introspective individual who actively tries to be in touch with their feelings and those of others. The participant also actively tries to take into account their limitations, energy levels, and capabilities when traveling or trying out new activities.

When traveling somewhere for work with their vehicle, this participant tends to learn the route by heart beforehand and seems to have a good sense of direction. Still, it was difficult to describe the exact turns, cues, and landmarks they use during route-following or other orientation-related tasks, even when prompted by the interviewer in several different ways. This participant talked mainly about their vision, and focused predominantly on mobility and safety issues (e.g., trying to detect oncoming cyclists, or avoiding collision with the sidewalk when cycling), social issues regarding navigation (e.g., seeing a person but not being able to recognize someone’s face), being hindered by vision-related issues like bright lights (e.g., again, in context of seeing oncoming traffic users), and by their own emotions during navigation (e.g., the learning process with regards to their attitude towards making mistakes, and feelings of responsibility as a visually impaired person on the road, or as a parent). The participant talked very little about using their other senses, such as hearing or touch, compared to other interviewees.

### Appendix B.8. Case 08

Participant 08 is the youngest participant (aged between 30–40 years old) and has been very severely blind since birth (diagnosis unknown). This participant has been using canes since early childhood; started out with a tap cane, though switched to a rolling tip cane around 24 years of age. This participant has studied higher vocational training, but currently does some impairment-related volunteering. This participant is a big soccer fan and visits soccer games with visually impaired friends in their free time. This participant can also read Braille. The interview took place at their apartment, at which they apologized for the dustiness. They came across as a spontaneous person who likes to make a lot of jokes about both lighthearted and serious topics.

In terms of orientation, this participant has a self-reported preference for their sense of touch. This is also reflected in how much they talked about using touch, and in how often they mentioned the need for more shorelines or directional tactile paving. This participant talked notably less about their knowledge of environments, suggesting that they might not tap into their survey knowledge consciously or frequently. This is supported by the participant mentioning that a friend of theirs, who is congenitally blind, also told they have no sense of direction. However, this participant does use smartphone apps for navigation and mentioned liking it when the app would tell them which stores or restaurants they are passing by. This implies that this participant does value having knowledge of certain landmarks within an environment. In terms of attitudes, their focus was on their emotions and needs (e.g., explicitly telling stories about moments or locations where they encounter issues), as well as the interpersonal dynamics during wayfinding (e.g., fun interactions during wayfinding, feeling like a burden to others, concerns about looking silly while searching for a certain landmark, or being pranked by a visually impaired friend who startled them by suddenly grabbing them at the bus stop). This participant also mentioned asking passersby for help or walking along with friends a lot, which made sense in the context of their sociable nature.

### Appendix B.9. Case 09

Participant 09 (aged between 60–70 years old) became moderately visually impaired around age 55 and has been using a white cane since. Their visual impairment resulted from a viral infection due to an immune disease (right eye: vasculitis; left eye: retinal detachment and macula damage). Participant 09 came across as an amicable and earnest person with an expressive way of talking. On the one hand, they would describe how “sick and tired” they could get because of issues concerning accessibility. On the other hand, this participant would describe how beautiful the orientation and mobility training was for them, and how they “would never forget it”. The participant told the interviewer how anxious they initially were when navigating and how the training helped them gain confidence and independence. The participant likes to try many things autonomously and keep challenging themselves, and only looks for solutions elsewhere as a last resort. The interview took place at the Utrecht Science Park, which the participant preferred, so they could practice their knowledge of traveling through Utrecht Central Station.

Participant 09 often elaborated on how their partner, who is also visually impaired, navigates. The participant would tell about the challenges their partner encounters and how they differ from those they themselves face. The participant talked a lot about traveling together with their partner, how it is both beneficial and pleasant. It seems like the participant uses their knowledge of an environment, and often mentions shops when describing their routes. Lastly, this participant talked about preferring their residual vision, for example to detect landmarks or to find fine-grained navigational goals. An example of this is seeing the bright red lights of a kiosk’s store name at Utrecht Central Station to orient themselves.

### Appendix B.10. Case 10

Participant 10 (aged between 50–60 years old) suffers from retinitis pigmentosa and has been visually impaired since birth. The impairment has worsened progressively over their lifespan, and the participant is currently very severely visually impaired. They have been using a white cane for about 23 years and have also been traveling with a guide dog for about 14 years at the time of the interview. Additionally, the participant wears a cap and sunglasses when traveling outdoors. The participant came across as a sympathetic, yet somewhat serious person who lives an active lifestyle. In their daily life, they do impairment-related volunteering and have several hobbies (running, and spending time ‘in the digital world’). The interview took place at the participant’s home.

This person described alternative routes and reasons for choosing one over the other, though they would be able to take both of these options. This is one expression of how they used knowledge of the environment to navigate (e.g., the participant talked relatively often about estimations of distance and time until a certain point along the route, suggesting a pretty accurate map of the environment in mind when navigating), focusing on both orientation and mobility. Their focus was also on their various wayfinding preferences. Furthermore, this participant talks about using the guide dog to find fine-grained navigational goals, but also talks a lot about the fact that dogs can make mistakes too, and also kept explicitly mentioning that they also check the landmarks using the cane whenever giving the dog a command. This might be another expression of their need for efficiency, which also became apparent from their preference to maintain a fast walking pace.

### Appendix B.11. Case 11

Participant 11 (between 50–60 years old) has been moderately visually impaired for three years at the time of the interview due to conical dystrophy. When traveling outdoors, this person wears a hat and sunglasses and does not have a guide dog or a cane. In their daily life, this participant is a stay-at-home parent and does some volunteering. The interview took place at a café at Utrecht Central Station, due to issues with public transport to the Utrecht Science Park.

This person, like participants 07 and 09, also talked relatively often about using their residual vision, remarking more than once that they see themselves as a “fake visually impaired person” because they still use the sense of sight. Their focus was undoubtedly on mobility, and specifically on tripping hazards, safety, other traffic users (similar to 07), and obstacles. Namely, the participant talked about various situations in which they encountered objects that caused or might cause harm (chains between bollards, street planters, holes in the road, unexpected curbs, barbed wire, et cetera). They did not talk much at all about using other senses like hearing or touch (also like 07). Remarkably, they rarely talked about asking for help. When asked about this by the interviewer, this participant answered that they are indeed a bit reserved when it comes to asking help because he feels that it is easier to do it yourself when you are well-prepared and that it might also stem from a need for security, and a need to feel in control. However, a need for independence or control was not explicitly mentioned otherwise.
